# Sleep Duration and Stroke: A Mendelian Randomization Study

**DOI:** 10.3389/fneur.2020.00976

**Published:** 2020-10-07

**Authors:** Hui Lu, Peng-Fei Wu, Rui-Zhuo Li, Wan Zhang, Guo-xiang Huang

**Affiliations:** ^1^Department of Neurology, Xuanwu Hospital, Capital Medical University, Beijing, China; ^2^Center for Medical Genetics & Hunan Provincial Key Laboratory for Medical Genetics, School of Life Sciences, Central South University, Changsha, China; ^3^Department of Neurology, Beth Israel Deaconess Medical Center & Harvard Medical School, Boston, MA, United States; ^4^School of Medicine, South China University of Technology, Guangzhou, China; ^5^Biology Department, College of Arts & Sciences, Boston University, Boston, MA, United States; ^6^Department of Neurology, Affiliated Hospital of Nantong University, Nantong, China

**Keywords:** stroke, sleep duration, mendelian randomization, genome-wide association study, single nucleotide polymorphism

## Abstract

**Study Objectives:** To clarify the effects of sleep duration on stroke and stroke subtypes, we adopted a Mendelian randomization (MR) approach to evaluate their causal relationship.

**Methods:** A genome-wide association study including 446,118 participants from UK biobank was used to identify instruments for short sleep, long sleep and sleep duration. Summary-level data for all stroke, ischemic stroke, intracerebral hemorrhage, and their subtypes were obtained from meta-analyses conducted by the MEGASTROKE consortium. MR analyses were performed using the inverse-variance-weighted method, weighted median estimator, MR pleiotropy residual sum and outlier (MR-PRESSO) test, and MR-Egger regression. Sensitivity analyses were further performed using leave-one-out analysis, MR-PRESSO global test and Cochran's Q test to verify the robustness of our findings.

**Results:** By two-sample MR, we didn't find causal associations between sleep duration and risk of stroke. However, in the subgroup analysis, we found weak evidence for short sleep in increasing risk of cardio-embolic stroke (odds ratio [OR], 1.33; 95% confidence interval [CI], 1.11–1.60; *P* = 0.02) and long sleep in increasing risk of large artery stroke [OR, 1.41; 95% CI, 1.02–1.95; *P* = 0.04]. But the associations were not significant after Bonferroni correction for multiple comparisons.

**Conclusions:** Our study suggests that sleep duration is not causally associated with risk of stroke and its subtypes.

## Introduction

Stroke is the second leading cause of death worldwide, with an age-standardized mortality rate of 86.5 per 100,000 population per year (1). Although stroke incidence, prevalence, mortality and disability-adjusted life-years rates have declined from 1990 to 2017, the absolute number of people who developed new stroke, died, survived or remained disabled from stroke has almost doubled (2). Globally in 2017, ischemic stroke constituted 65%, primary intracranial hemorrhage (ICH)-26% and subarachnoid hemorrhage-9% of all incidents of stroke (2). Given the global burden of stroke, it is imperative to identify other modifiable lifestyle factors to prevent stroke.

Sleep is gaining recognition as an important lifestyle factor relevant to the prevention of stroke. A large proportion of people sleep less or more than the recommended 7–8 h of sleep (3). Recent reports have suggested an association between both short (<6 h) and long (>9 h) sleep duration and risk of stroke (4, 5). However, the evidence supporting this association is not consistent. Some studies have found a link between long or short sleep duration and an increased risk of stroke (6–9), whereas other studies have claimed no significant association (10, 11). These conflicting findings from observational epidemiological studies may be due to differences in the study population, sample size, sleep duration assessment methods, or covariates adjusted in the statistical models. Also, observational studies are prone to biases, particularly from confounders such as disturbed sleep conditions and poor health. It is also difficult to distinguish if abnormal sleep duration causes stroke, or risk factors like poor health result in both abnormal sleep duration and stroke. In addition, whether sleep duration has different influences on different stroke subtypes is unknown.

These limitations may be overcome by use of genetic proxies of lifetime exposure in Mendelian randomization (MR) (12). While observational studies investigate the association between an exposure and outcome, MR investigates the link between genetic variants relating to the exposure and outcome (13). By studying the genetic variants rather than the exposure directly, MR overcomes the potential impact of confounding factors on the exposure (13). Therefore, MR has advantages in its ability to draw conclusions about the causal relationship between exposure and outcome.

Sleep duration is a heritable trait. Twin- and family- based studies have suggested that 9 ~ 45% of variability in self-reported sleep duration is influenced by genetic factors (14, 15). Recent genome-wide association studies (GWAS) in up to 446,118 participants from the UK Biobank identified 78 single nucleotide polymorphism (SNP) for habitual self-reported sleep duration (15). In this study, we utilized a two-sample MR approach to investigate whether there existed causal effects of lifetime sleep duration on stroke or stroke subtypes.

## Methods

This study was based on MR design, which utilized publicly shared databases; no additional participant consent is required.

### GWAS Data Sources

Summary statistics for sleep traits were obtained from the Sleep Disorder Knowledge Portal (http://sleepdisordergenetics.org/). Dashti et al. has performed the most up-to-data GWAS meta-analysis of self-reported habitual short sleep (<7 h/days; 106,192 individuals), long sleep (≥9 h/day; 34,184 individuals), and sleep duration in 446,118 European-ancestry adults from the UK Biobank (15). Seventy-eight loci genome-wide significantly associated with sleep duration (*P* < 5 × 10^−8^), 27 and eight loci for short and long sleep in terms of 7–8 h sleep duration (305,742 controls), respectively, were identified.

Stroke datasets were released by the MEGASTROKE consortium (http://www.megastroke.org/). The results for GWAS meta-analysis restricted to European participants (40,585 cases and 406,111 controls) were available. Stroke were categorized into any ischemic stroke (AIS; 34,217 cases), large artery stroke (LAS; 4,373 cases), cardioembolic stroke (CES; 7,193 cases), and small vessel stroke (SVS; 5,386 cases) (16). Summary statistics for ICH were obtained from another meta-analysis by the International Stroke Genetics Consortium. 1,545 cases defined by neurological deficits with compatible brain imaging and 1,481 controls of European ancestry were incorporated (17). ICH was subtyped into lobar (the lesion originating at the cerebral cortex or cortical-subcortical junction; 686 cases), and non-lobar (thalamus, internal capsule, basal ganglia, deep periventricular white matter, cerebellum, or brain stem; 909 cases). All ethical approval had been obtained in these original researches.

### Selection of Instrumental SNPs

MR analyses were based on genetic instrumental variables, a set of independent SNPs (*r*^2^ < 0.1 in European populations, 1000 Genomes Project Phase 3) significantly associated with the exposure, for which either their own or their proxy SNPs (*r*^2^ > 0.8) outcome association statistics should be available concurrently. For sleep-stroke MR analyses, among SNPs associated with short sleep, long sleep and sleep duration, rs142180737, rs549961083, and rs2139261, respectively, was removed owing to no available proxies. For sleep-ICH analyses, 18 short sleep-related SNPs were retained, among which 3 proxies (rs12705966, rs7549825, rs11955683; *r*^2^ ≥ 0.86, D′ ≥ 0.97) were adopted; 5 long sleep-related SNPs were retained, and rs17688916 was proxied by rs113871181 (*r*^2^ = 0.90, D′ = 0.99); 61 sleep duration-related SNPs were retained, among which 8 proxies (rs4470910, rs62535668, rs1517564, rs77493530, rs7549825, rs3786694, rs67008484, rs180767; *r*^2^ ≥ 0.82, D′ ≥ 0.96) were utilized.

### Mendelian Randomization Analysis

We performed a two-sample MR using R (version 3.5.3) and *TwoSampleMR* and *MR-PRESSO* packages (18, 19). Firstly, an MR estimate for each instrumental SNP was generated using the Wald method (20, 21). To compute the overall estimate, the inverse-variance weighted (IVW) model was used for the primary MR analysis. Three additional methods, the MR pleiotropy residual sum and outlier (MR-PRESSO), weighted median estimator (WM) and MR-Egger regression, based on different MR assumptions were conducted. IVW generally gave a consistent estimate of the causality, which was based on a fixed-effect meta-analysis. The inverse-variance weighted estimate may be biased, however, in the presence of invalid instrumental variables with horizontal pleiotropy (20). MR-PRESSO has the advantage of detecting horizontal pleiotropy and yielding a corrected estimate via outlier removal if necessary (18). WM and MR-Egger were less statistically well-powered, yet more robust to horizontal pleiotropy. The weighted median estimator pooled effects of individual variants efficiently under the prerequisite that more than 50% of the weight came from valid instrumental variables (22). MR-Egger regression assumed that the pleiotropic associations are independent and conducted a weighted linear regression of the outcome coefficient on the exposure coefficient. The intercept and slope of MR-Egger regression provided an exploration of pleiotropy and the causality estimate adjusted for pleiotropy (18). Accordingly, the comprehensive evaluation of MR results was rendered possible through 4 MR approaches based on different assumptions.

Furthermore, 3 sensitivity analyses were implemented. To explore whether the MR estimates were disproportionately influenced by certain SNP alone, we performed a leave-one-out analysis by omitting instrumental SNPs one by one (23). MR-PRESSO global and distortion test was conducted likewise, detecting the significant distortion in the causal estimates before and after outlier removal. We also examined the heterogeneity across all instrumental SNPs quantified by Cochran's Q statistics.

## Results

Table 1 summarized the main results of the MR analyses exploring the causality between 3 self-reported sleep traits and stroke, ICH and their subtypes. Overall, genetically determined short sleep was not causally associated with stroke (odds ratio [OR], 0.91; 95% confidence interval [CI], 0.78–1.07) and ICH (OR, 0.55; 95% CI, 0.23–1.29). In subtype analyses, there was weak evidence showing that per unit increment in log-odds for short sleep would increase risk for CES by 33% (OR, 1.33; 95% CI, 1.11–1.60; *P* = 0.02).

**Table 1:** Mendelian randomization estimates for causal effect of sleep on ischemic stroke.

**Outcome and method**	**Short sleep (<7 h)**		**Long sleep (>9 h)**		**Total sleep duration (hours/day)**	
	**OR (95% CI)**	***P*-value**	**OR (95% CI)**	***P*-value**	**OR (95% CI)**	***P*-value**
**ALL STROKE**						
IVW^a^	0.91 (0.78–1.07)	0.25	1.13 (1.00–1.27)	0.05	0.99 (0.99–1.00)	0.13
MR–PRESSO^b^	0.91 (0.83–1.00)	0.11	1.13 (1.01–1.25)	0.04	0.99 (0.99–1.00)	0.14
WM^c^	0.93 (0.77–1.12)	0.45	1.12 (0.95–1.33)	0.18	0.99 (0.99–1.00)	0.35
**ANY ISCHEMIC STROKE**						
IVW	0.96 (0.82–1.12)	0.58	1.10 (0.97–1.26)	0.14	0.99 (0.99–1.00)	0.39
MR–PRESSO	0.96 (0.86–1.06)	0.43	1.10 (0.99–1.23)	0.10	0.99 (0.99–1.00)	0.39
WM	0.98 (0.80–1.19)	0.82	1.05 (0.88–1.26)	0.58	0.99 (0.99–1.00)	0.85
**LARGE ARTERY STROKE**						
IVW	1.26 (0.85–1.86)	0.26	1.41 (1.02–1.95)	0.04	0.99 (0.99–1.00)	0.15
MR–PRESSO	1.26 (0.92–1.71)	0.20	1.41 (1.02–1.95)	0.05	0.99 (0.99–1.00)	0.15
WM	1.15 (0.72–1.86)	0.55	1.41 (0.89–2.24)	0.14	0.99 (0.99–1.00)	0.77
**CARDIOEMBOLIC STROKE**						
IVW	1.33 (0.98–1.81)	0.07	0.99 (0.74–1.32)	0.94	1.00 (0.99–1.00)	0.49
MR–PRESSO	1.33 (1.11–1.60)	0.02	0.99 (0.74–1.32)	0.94	1.00 (0.99–1.00)	0.49
WM	1.26 (0.86–1.84)	0.24	0.74 (0.52–1.07)	0.11	1.00 (0.99–1.01)	0.33
**SMALL VESSEL STROKE**						
IVW	0.86 (0.56–1.34)	0.51	1.02 (0.75–1.37)	0.91	0.99 (0.99–1.00)	0.38
MR–PRESSO	0.86 (0.56–1.34)	0.53	1.02 (0.76–1.37)	0.91	0.99 (0.99–1.00)	0.38
WM	0.97 (0.60–1.55)	0.89	0.99 (0.64–1.51)	0.95	0.99 (0.99–1.00)	0.57
**ALL ICH**						
IVW	0.55 (0.23–1.29)	0.17	1.20 (0.44–3.26)	0.72	1.00 (0.99–1.00)	0.94
MR–PRESSO	1.00 (1.00–1.00)	0.48	1.20 (0.44–3.26)	0.73	1.00 (0.99–1.00)	0.94
WM	0.40 (0.14–1.18)	0.10	2.01 (0.64–6.32)	0.23	0.99 (0.99–1.00)	0.89
**LOBAR ICH**						
IVW	0.40 (0.13–1.22)	0.11	1.18 (0.42–3.30)	0.76	1.00 (0.99–1.00)	0.54
MR–PRESSO	0.40 (0.20–0.80)	0.06	1.18 (0.43–3.18)	0.75	1.00 (0.99–1.00)	0.48
WM	0.32 (0.08–1.21)	0.09	1.02 (0.24–4.39)	0.98	0.99 (0.99–1.00)	0.90
**NON–LOBAR ICH**						
IVW	0.63 (0.23–1.75)	0.38	1.25 (0.39–4.00)	0.71	0.99 (0.99–1.00)	0.52
MR–PRESSO	0.63 (0.27–1.49)	0.36	1.25 (0.39–4.00)	0.71	0.99 (0.99–1.00)	0.52
WM	0.50 (0.15–1.66)	0.26	1.25 (0.32–4.99)	0.75	0.99 (0.99–1.00)	0.93

a *IVW, inverse-variance weighted analysis*.

b *MR-PRESSO, Mendelian randomization pleiotropy residual sum and outlier method*.

c *WM, weighted median estimator*.

MR results did not support the causal association between long sleep and stroke (OR, 1.13; 95% CI, 1.00–1.27) or ICH (OR, 1.20; 95% CI, 0.44–3.26), either. But there was suggestive evidence supporting long sleep as a possible risk factor for LAS. The OR of LAS per unit increase in log-odds for long sleep was 1.41 (95% CI, 1.02–1.95; *P* = 0.04). The causality between short or long sleep and the other subtypes of stroke was not statistically significant. Besides, no causal effects of sleep duration on stroke, ICH, or their subtypes were identified.

The MR-Egger regression and MR-PRESSO global tests demonstrated no presence of horizontal pleiotropy among all instrumental SNPs (Supplementary Tables 1–3). The leave-one-out analyses and Cochran's Q tests indicated no heterogeneity (Supplementary Figures 1–3). Notably, the results from different MR methods were consistent on the whole, yet we had inadequate power to detect weak associations, that is, an estimated statistical power below 80% to detect the true effect size between 0.8 and 1.2 (Supplementary Table 4).

## Discussion

In this study, we have not found any evidence that sleep traits (short sleep, long sleep, or total sleep duration) were causally associated with the risk of stroke or stroke subtypes (AIS, SVS, or ICH). However, we found weak evidence that short sleep can increase the risk of CES and long sleep can increase the risk of LAS, albeit the associations did not reach statistical significance after correction for multiple comparisons. To our knowledge, no previous MR study had assessed the causal association of sleep duration and risk of stroke subtypes (24).

Lots of population-based cohort studies have been performed examining the association of sleep duration and stroke. However, results of meta-analyses based on these cohort studies are conflicting. One meta-analysis including 19 studies (31 cohorts) with a total of 816,995 individuals shows both short (risk ratio [RR], 1.32; 95% CI, 1.18–1.47) and long (RR, 1.48; 95% CI, 1.31–1.68) sleep can increase the risk of stroke (9). However, another meta-analysis including 153 studies with 5,172,710 participants found no sufficient evidence on the association between short sleep and risk of stroke (RR, 1.08; 95% CI, 0.98–1.19) (11). To clarify their relationship, we leveraged pre-existing databases from the UKB and MEGASTROKE projects in this study and concluded no significant association between sleep duration and stroke.

Different subtypes of stroke have different pathological processes, so we went further to investigate the effects of sleep duration upon subtypes of stroke. In the subgroup analysis of our MR study, we found weak evidence for long sleep in increasing risk of LAS. This result seemed consistent with earlier MR study on myocardial infarction (25). A potential explanation is that long sleep might be associated with an increased risk of atherosclerosis (26). Spending excessive time in bed can elicit daytime lethargy and exacerbate sleep fragmentation (27), which was considered to be associated with more severe arteriolosclerosis and subcortical macroscopic infarcts (4). In addition, long sleep duration may be linked to low levels of physical activity, type 2 diabetes, poor sleep quality, poor physical/mental health, low socioeconomic status, and vary by ethnicity (28–32). All of these factors might increase the risk of LAS. We also found weak evidence for short sleep in increasing risk of CES. The possible hypothesis is that short sleep duration is independently associated with prevalent and incident atrial fibrillation (AF) (33). Each 1-h reduction in sleep duration was associated with 17% greater risk of prevalent AF and 9% greater risk of incident AF (33). Sleep deprivation increases atrial electromechanical delay, increases sympathetic nervous system activity and activates pro-inflammatory systems, which are pathways predisposing to AF (34–36). AF has been well-acknowledged as the leading cause for CES, which may explain the weak association of short sleep with increased risk of CES in our study. Lastly, sleep duration is merely one of the commonly reported sleep traits. We did not investigate the causal effect of other sleep characteristics on risk of stroke, such as sleep apnea and REM percentage. Long sleep duration is reported to be associated with an increased risk of stroke in obstructive sleep apnea patients with existing cardiovascular disease (37). Meanwhile, a recent study has showed that less REM sleep was associated with greater risk of cardiovascular mortality in middle-aged and older adults (38). Future MR studies are warranted to explore the role of more detailed sleep characteristics.

There were several limitations for our study. Firstly, we could not detect a non-linear relationship between sleep duration and stroke using these MR methods and summary-level statistics. Secondly, intrinsic pleiotropy of instrumental SNPs could hinder the reliability of MR results (39); nevertheless, we failed to detect any horizontal pleiotropy when employing several efficient tools to scrutinize it in this study. Thirdly, SNPs identified by GWAS are acknowledged to explain a small proportion of variance, including the sleep traits-associated SNPs; hence, this study had restricted power to detect a weak effect, especially the association of sleep traits with ICH, considering the much smaller sample size as opposed to other subtypes of stroke. Fourthly, data on sleep duration are self-reported by the participants which might be less accurate than objective methods, such as polysomnography. Concerns about self-reported sleep duration were raised, and acceptable correlations between self-reported sleep duration and that measured by actigraphy have been reported (40). More detailed characteristics for sleep duration were not collected, such as chronotype, napping, dozing and snoring, which may undermine this study's clinical implications. Fifthly, our database originated from European-ancestry studies, whereas it is well-known that stroke burden varies among ethnicities worldwide, such as in the Chinese Han population, in which stroke seems more prevalent than in Europeans (41); therefore, we should be cautious when generalizing the conclusion to other ethnicities worldwide.

In summary, our study found little evidence on causal relationship between genetically predicted sleep duration with risk of stroke and its subtypes.

## Data Availability Statement

All datasets generated for this study are included in the article/Supplementary Material.

## Author Contributions

HL: conceptualization, methodology, writing-original draft preparation, writing-reviewing and editing, and supervision. P-FW: conceptualization, data curation, software, formal analysis, writing-original draft preparation, and data visualization. R-ZL: data curation, software, and formal analysis. WZ: writing-reviewing and editing, data visualization, and validation. GH: validation, resources, writing-reviewing, editing, and supervision. All authors contributed to the article and approved the submitted version.

## Conflict of Interest

The authors declare that the research was conducted in the absence of any commercial or financial relationships that could be construed as a potential conflict of interest.
