# Supplementary material for: Sleep Duration and Stroke: A Mendelian Randomization Study
Source: Front Neurol. 2020 Oct 7;11:976. doi: 10.3389/fneur.2020.00976 (PMC7575720; doi:10.3389/fneur.2020.00976)
Supplement: Supplementary file 1 [file Data_Sheet_1.PDF]

Supplementary Content

Table S1. Sensitivity Analysis Results for the MR Analyses of Short Sleep on Stroke 2

Table S2. Sensitivity Analysis Results for the MR Analyses of Long Sleep on Stroke 3

Table S3. Sensitivity Analysis Results for the MR Analyses of Sleep Duration on Stroke 4

Table S4. Statistical power rough estimations for the Mendelian randomization analyses 5

Table S5. Harmonised Datasets for the MR Analyses of sleep exposures on all Stroke 7

Table S6. Harmonised Datasets for the MR Analyses of sleep exposures on any ischemic Stroke 12

Table S7. Harmonised Datasets for the MR Analyses of sleep exposures on large artery Stroke 17

Table S8. Harmonised Datasets for the MR Analyses of sleep exposures on cardioembolic stroke 22

Table S9. Harmonised Datasets for the MR Analyses of sleep exposures on small vessel stroke 27

Table S10. SNP Proxies Utilized in the MR analyses of Sleep and ICH 32

Table S11. Harmonised Datasets for the MR Analyses of Short Sleep on ICH33

Table S12. Harmonised Datasets for the MR Analyses of Long Sleep on ICH34

Table S13. Harmonised Datasets for the MR Analyses of Sleep Duration on ICH 35

Fig S1. Causal effect of long sleep on large artery stroke by individual SNP and primary overall estimate by Mendelian randomization. 38

Fig S2. Causal effect of short sleep on cardioembolic stroke by individual SNP primary overall estimate by Mendelian randomization. 39

Fig S3. Leave-one-out Plots for the MR Analyses of Short Sleep on Stoke 40

Fig S4. Leave-one-out Plots for the MR Analyses of Long Sleep on Stoke 41

Fig S5. Leave-one-out Plots for the MR Analyses of Sleep Duration on Stoke 42

Table S1. Sensitivity Analysis Results for the MR Analyses of Short Sleep on Stroke

	MR-Egger Test			PRESSO Global Test		Cochrane Q Test		
	**Intercept**	**Se**	**Pval**	**RSSobs**	**Pval**	**Q**	**Q_df**	**Pval**
All Stroke	0.005	0.008	0.566	20.997	0.777	19.637	25	0.766
Any ischemic stroke	-0.002	0.009	0.813	19.498	0.844	18.145	25	0.836
Large artery stroke	-0.011	0.022	0.618	28.372	0.448	26.469	26	0.438
Cardioembolic stroke	-0.008	0.021	0.705	34.881	0.137	32.198	25	0.152
Small vessel stroke	0.015	0.021	0.480	25.996	0.492	24.191	25	0.508
Any ICH	0.124	0.074	0.113	30.342	0.074	27.265	17	0.054
Lobar ICH	0.115	0.088	0.210	28.609	0.083	25.943	17	0.076
Non-Lobar ICH	0.135	0.080	0.109	17.693	0.541	15.818	17	0.537

Table S2. Sensitivity Analysis Results for the MR Analyses of Long Sleep on Stroke

	MR-Egger Test			PRESSO Global Test		Cochrane Q Test		
	**Intercept**	**Se**	**Pval**	**RSSobs**	**Pval**	**Q**	**Q_df**	**Pval**
All Stroke	-0.008	0.014	0.612	2.459	0.896	1.804	5	0.876
Any ischemic stroke	-0.006	0.015	0.719	3.255	0.886	2.617	6	0.855
Large artery stroke	-0.047	0.038	0.278	4.854	0.728	3.693	6	0.718
Cardioembolic stroke	-0.011	0.030	0.741	2.823	0.922	2.068	6	0.913
Small vessel stroke	-0.010	0.047	0.838	12.237	0.168	8.856	6	0.182
Any ICH	0.074	0.118	0.573	4.920	0.554	3.316	4	0.506
Lobar ICH	0.043	0.140	0.780	4.335	0.606	2.858	4	0.582
Non-Lobar ICH	0.118	0.154	0.498	2.316	0.841	1.570	4	0.814

Table S3. Sensitivity Analysis Results for the MR Analyses of Sleep Duration on Stroke

	MR-Egger Test			PRESSO Global Test		Cochrane Q Test		
	**Intercept**	**Se**	**Pval**	**RSSobs**	**Pval**	**Q**	**Q_df**	**Pval**
All Stroke	-0.002	0.005	0.743	92.361	0.137	90.158	76	0.128
Any ischemic stroke	-0.001	0.005	0.846	88.322	0.195	86.120	76	0.200
Large artery stroke	0.006	0.014	0.668	95.711	0.098	93.222	76	0.087
Cardioembolic stroke	-0.009	0.010	0.359	83.232	0.354	81.100	76	0.323
Small vessel stroke	-0.008	0.013	0.538	92.717	0.116	90.537	76	0.122
Any ICH	0.036	0.030	0.246	4.920	0.554	65.860	60	0.281
Lobar ICH	0.022	0.038	0.563	70.646	0.190	68.457	59	0.187
Non-Lobar ICH	0.038	0.038	0.327	45.591	0.920	44.040	59	0.927

Table S4. Statistical power rough estimations for the Mendelian randomization analyses

Exposure	Outcome	Instrumental variable	Outcome sample size	Cases/controls	Percentage of variance explained by instrumental variables (%)	Given Power	Significance level	Odds ratio (Upper)	Odds ratio (Lower)
Short Sleep	All Stroke	26 SNPs	446,696	40,585/406,111	1.20%	80%	0.05	1.135	0.869
Short Sleep	Any ischemic stroke	26 SNPs	440,328	34,217/406,111	1.20%	80%	0.05	1.146	0.857
Short Sleep	Large artery stroke	27 SNPs	410,484	4,373/406,111	1.20%	80%	0.05	1.390	0.611
Short Sleep	Cardioembolic stroke	26 SNPs	413,304	7,193/406,111	1.20%	80%	0.05	1.306	0.699
Short Sleep	Small vessel stroke	26 SNPs	411,497	5,386/406,111	1.20%	80%	0.05	1.354	0.650
Short Sleep	Any ICH	18 SNPs	3,026	1,545/1,481	0.80%	80%	0.05	2.989	0.340
Short Sleep	Lobar ICH	18 SNPs	2,167	686/1,481	0.80%	80%	0.05	2.950	0.130
Short Sleep	Non-Lobar ICH	18 SNPs	2390	909/1,481	0.80%	80%	0.05	2.940	0.220
Long Sleep	All Stroke	7 SNPSs	446,696	40,585/406,111	0.95%	80%	0.05	1.151	0.853
Long Sleep	Any ischemic stroke	7 SNPSs	440,328	34,217/406,111	0.95%	80%	0.05	1.163	0.840
Long Sleep	Large artery stroke	7 SNPSs	410,484	4,373/406,111	0.95%	80%	0.05	1.438	0.569
Long Sleep	Cardioembolic stroke	7 SNPSs	413,304	7,193/406,111	0.95%	80%	0.05	1.344	0.660
Long Sleep	Small vessel stroke	7 SNPSs	411,497	5,386/406,111	0.95%	80%	0.05	1.395	0.610
Long Sleep	Any ICH	5 SNPs	3,026	1,545/1,481	0.68%	80%	0.05	3.300	0.317
Long Sleep	Lobar ICH	5 SNPs	2,167	686/1,481	0.68%	80%	0.05	3.110	0.095
Long Sleep	Non-Lobar ICH	5 SNPs	2390	909/1,481	0.68%	80%	0.05	3.150	0.185
Sleep Duration	All Stroke	77 SNPSs	446,696	40,585/406,111	0.66%	80%	0.05	1.183	0.823

Sleep Duration	Any ischemic stroke	77 SNPSs	440,328	34,217/406,111	0.66%	80%	0.05	1.198	0.810
Sleep Duration	Large artery stroke	77 SNPSs	410,484	4,373/406,111	0.66%	80%	0.05	1.525	0.480
Sleep Duration	Cardioembolic stroke	77 SNPSs	413,304	7,193/406,111	0.66%	80%	0.05	1.410	0.595
Sleep Duration	Small vessel stroke	77 SNPSs	411,497	5,386/406,111	0.66%	80%	0.05	1.474	0.530
Sleep Duration	Any ICH	61 SNPSs	3,026	1,545/1,481	0.52%	80%	0.05	3.800	0.275
Sleep Duration	Lobar ICH	61 SNPSs	2,167	686/1,481	0.52%	80%	0.05	3.450	0.042
Sleep Duration	Non-Lobar ICH	61 SNPSs	2390	909/1,481	0.52%	80%	0.05	3.490	0.140

Table S5. Harmonised Datasets for the MR Analyses of sleep exposures on all Stroke

exposure	SNP	Chr:Pos	EA	OA	Association with exposures				All stroke			
					EAF	Beta	Se	Pval	EAF	Beta	Se	Pval
Short sleep	rs2863957	2:114089551	A	C	0.782	0.054	0.007	2.60E-18	0.226	-0.006	0.011	0.576
	rs13107325	4:103188709	T	C	0.075	0.075	0.011	2.50E-13	0.073	-0.001	0.020	0.959
	rs1229762	7:114218582	T	C	0.665	0.037	0.006	1.10E-12	0.656	0.000	0.010	0.985
	rs1380703	2:57941287	A	G	0.384	0.035	0.006	1.60E-11	0.619	-0.007	0.010	0.504
	rs12963463	18:53099093	T	C	0.299	0.029	0.006	1.90E-11	0.705	0.007	0.010	0.483
	rs75539574	2:58871658	A	C	0.915	0.045	0.011	8.40E-11	0.912	0.012	0.017	0.483
	rs17388803	15:48027204	A	C	0.106	0.053	0.010	6.50E-10	0.879	-0.023	0.015	0.119
	rs4585442	5:135508381	A	G	0.311	0.031	0.006	8.10E-10	0.698	-0.004	0.010	0.702
	rs1607227	11:28808617	T	G	0.705	0.031	0.007	1.50E-09	0.299	0.005	0.010	0.656
	rs2820313	1:201870221	A	G	0.341	0.031	0.006	2.30E-09	0.672	-0.017	0.010	0.087
	rs17005118	4:82288564	A	G	0.265	0.030	0.007	2.50E-09	0.269	0.010	0.010	0.354
	rs5757675	22:39838892	T	G	0.260	0.034	0.007	2.70E-09	0.723	0.001	0.010	0.917
	rs12567114	1:98527951	A	G	0.725	0.036	0.007	4.10E-09	0.280	0.004	0.011	0.674
	rs142180737	6:28344731	C	T	0.009	0.154	0.032	4.40E-09	NA	NA	NA	NA
	rs2186122	1:66470206	A	T	0.562	0.024	0.006	4.80E-09	0.436	-0.021	0.009	0.020
	rs11763750	7:2080114	A	G	0.814	0.035	0.008	5.10E-09	0.200	-0.010	0.012	0.377
	rs12518468	5:7249696	T	C	0.328	0.031	0.006	8.50E-09	0.677	-0.009	0.010	0.373

	rs9367621	6:55040290	A	T	0.431	0.024	0.006	1.60E-08	0.583	-0.015	0.009	0.100
	rs3776864	5:102327868	A	C	0.667	0.031	0.006	1.70E-08	0.696	0.001	0.010	0.913
	rs60882754	8:52886619	A	T	0.939	0.055	0.012	1.80E-08	0.947	0.009	0.021	0.661
	rs59779556	16:56227965	T	G	0.554	0.025	0.006	2.00E-08	0.539	0.003	0.009	0.730
	rs2014830	3:50172397	T	C	0.698	0.030	0.006	2.70E-08	0.317	-0.015	0.010	0.145
	rs205024	17:11227352	T	C	0.617	0.031	0.006	2.70E-08	0.394	-0.004	0.010	0.663
	rs12661667	6:41792545	T	C	0.263	0.028	0.007	2.80E-08	0.253	-0.012	0.010	0.241
	rs7939345	11:47980568	T	G	0.208	0.035	0.007	4.00E-08	0.208	-0.008	0.012	0.516
	rs9321171	6:129848635	T	C	0.540	0.031	0.006	4.20E-08	0.454	0.005	0.009	0.606
	rs7524118	1:34736052	T	C	0.708	0.030	0.006	4.90E-08	0.295	0.007	0.010	0.509
Long sleep	rs6737318	2:114083120	G	A	0.222	0.076	0.011	3.40E-13	0.775	0.005	0.011	0.630
	rs75458655	11:118115331	T	C	0.023	0.185	0.029	5.40E-12	0.023	0.008	0.039	0.836
	rs17817288	16:53807764	A	G	0.518	0.039	0.009	8.90E-09	0.507	-0.008	0.009	0.371
	rs549961083	5:58184093	T	C	0.001	0.534	0.117	9.60E-09	NA	NA	NA	NA
	rs3751046	11:122828342	G	A	0.147	0.070	0.013	2.00E-08	0.852	0.005	0.013	0.690
	rs7534398	1:7767464	A	T	0.201	0.047	0.012	2.10E-08	0.199	0.003	0.012	0.810
	rs10899257	11:76415209	A	G	0.144	0.068	0.013	4.60E-08	0.145	-0.018	0.013	0.178
	rs17688916	17:43778680	T	A	0.796	0.071	0.012	1.10E-11	0.207	0.012	0.017	0.471
Sleep duration	rs10173260	2:210377845	C	T	0.606	0.77	0.139	2.90E-08	0.389	0.004	0.009	0.647
	rs10421649	19:9942262	A	T	0.557	0.798	0.138	6.90E-09	0.519	0.004	0.010	0.677
	rs10483350	14:29816155	G	A	0.195	1.042	0.172	1.50E-09	0.801	-0.033	0.012	0.005
	rs1057703	11:122830251	G	T	0.147	1.164	0.192	1.10E-09	0.851	0.005	0.013	0.708
	rs10761674	10:64618340	C	T	0.477	0.74	0.136	4.20E-08	0.519	0.001	0.009	0.956
	rs10973207	9:37100525	T	G	0.158	1.226	0.187	6.00E-11	0.161	0.011	0.014	0.428

rs11190970	10:103128332	G	A	0.799	0.923	0.169	4.60E-08	0.208	0.000	0.012	0.986
rs112230981	3:55879269	A	G	0.95	1.892	0.314	2.20E-09	0.954	-0.048	0.024	0.042
rs113113059	6:43160375	T	C	0.78	0.968	0.164	8.40E-09	0.791	-0.011	0.011	0.334
rs114614603	6:28584775	A	G	0.72	1.015	0.151	2.30E-11	0.746	0.003	0.015	0.834
rs11567976	5:137654218	T	C	0.571	0.768	0.137	2.10E-08	0.551	-0.015	0.009	0.099
rs11602180	11:48162453	C	T	0.837	1.095	0.184	2.30E-09	0.168	-0.005	0.013	0.686
rs11614986	12:110007939	A	G	0.821	0.983	0.177	2.70E-08	0.823	0.004	0.012	0.761
rs11621908	14:78495761	C	T	0.917	1.446	0.25	5.60E-09	0.085	0.000	0.017	0.996
rs11643715	16:23909538	G	C	0.291	0.834	0.15	3.20E-08	0.712	-0.007	0.010	0.483
rs117527039	17:44083402	G	A	0.774	0.994	0.163	1.00E-09	0.225	0.016	0.015	0.294
rs11885663	2:166944004	T	C	0.248	0.973	0.157	8.60E-10	0.257	0.020	0.010	0.051
rs12246842	10:21830580	A	G	0.46	0.804	0.136	3.90E-09	0.451	0.008	0.009	0.385
rs12567114	1:98527951	A	G	0.276	0.89	0.152	4.30E-09	0.280	0.004	0.011	0.674
rs12607679	18:53059748	T	C	0.738	1.208	0.156	8.30E-15	0.749	0.002	0.011	0.866
rs12611523	2:139195328	A	G	0.545	0.758	0.137	3.10E-08	0.552	0.010	0.009	0.285
rs1263056	11:116576415	A	G	0.519	0.768	0.137	2.00E-08	0.506	0.000	0.010	0.990
rs12791153	11:80685181	T	A	0.081	1.413	0.253	1.90E-08	0.915	0.008	0.018	0.662
rs13088093	3:135838598	G	T	0.336	0.976	0.144	7.00E-12	0.666	0.016	0.010	0.097
rs13109404	4:102896591	T	G	0.928	1.872	0.264	1.40E-12	0.937	-0.007	0.022	0.762
rs151014368	5:176751059	A	G	0.206	0.966	0.169	9.10E-09	0.230	-0.010	0.011	0.381
rs1517572	11:28829882	C	A	0.581	0.879	0.138	1.50E-10	0.419	0.001	0.010	0.962
rs1553132	11:88297740	G	A	0.258	0.87	0.155	2.50E-08	0.741	0.018	0.011	0.081
rs17427571	4:82254908	A	G	0.684	0.83	0.146	1.30E-08	0.673	0.009	0.010	0.384
rs174560	11:61581764	C	T	0.314	0.815	0.146	2.80E-08	0.691	0.024	0.010	0.021
rs17732997	3:70470834	C	G	0.569	0.776	0.137	1.20E-08	0.581	0.001	0.009	0.912
rs1776776	9:140497072	T	C	0.874	1.198	0.205	4.90E-09	0.860	0.003	0.014	0.847

rs180769	5:135615615	T	C	0.425	0.763	0.138	2.30E-08	0.436	0.003	0.010	0.785
rs1939455	11:101520886	G	T	0.879	1.226	0.214	1.20E-08	0.115	-0.017	0.015	0.271
rs205024	17:11227352	T	C	0.384	0.83	0.14	3.90E-09	0.394	-0.004	0.010	0.663
rs2072727	20:43538733	T	C	0.436	0.795	0.137	7.90E-09	0.429	-0.007	0.009	0.441
rs2079070	7:114126432	C	G	0.265	1.053	0.154	7.50E-12	0.276	-0.007	0.012	0.521
rs2139261	17:21313223	G	C	0.749	1.122	0.174	8.50E-11	NA	NA	NA	NA
rs2192528	4:18327896	A	G	0.48	0.802	0.136	2.70E-09	0.474	-0.006	0.009	0.505
rs2231265	6:89790201	G	A	0.772	0.897	0.162	2.70E-08	0.228	-0.008	0.011	0.450
rs269054	1:57864304	A	T	0.422	0.819	0.138	2.10E-09	0.418	-0.001	0.009	0.909
rs3095508	16:6550400	C	A	0.594	0.921	0.138	3.10E-11	0.385	-0.009	0.010	0.370
rs330088	8:9149746	C	T	0.547	0.868	0.137	2.70E-10	0.470	0.011	0.010	0.253
rs34354917	12:38764559	C	A	0.71	0.825	0.15	3.90E-08	0.286	0.004	0.011	0.699
rs34731055	7:2106928	T	C	0.181	1.168	0.177	3.70E-11	0.201	-0.004	0.012	0.734
rs35531607	4:92533225	C	T	0.474	0.77	0.136	1.50E-08	0.537	0.005	0.009	0.577
rs365663	5:1428883	A	G	0.546	0.878	0.137	1.00E-10	0.543	0.002	0.009	0.833
rs374153	2:40382712	C	T	0.158	1.057	0.186	9.10E-09	0.848	-0.007	0.013	0.578
rs4128364	2:147612734	C	T	0.339	0.876	0.143	1.40E-09	0.653	0.029	0.010	0.003
rs4538155	2:157040773	T	C	0.647	0.779	0.142	3.60E-08	0.653	0.017	0.010	0.082
rs4592416	11:43800474	G	A	0.464	0.881	0.136	9.30E-11	0.544	0.024	0.009	0.008
rs460692	5:3126584	C	T	0.137	1.263	0.2	3.60E-10	0.853	-0.008	0.014	0.554
rs4767550	12:117951150	G	A	0.414	0.858	0.139	6.30E-10	0.583	-0.002	0.009	0.816
rs55658675	14:65554638	C	T	0.645	0.788	0.142	2.00E-08	0.347	-0.002	0.010	0.856
rs56372231	5:102321905	T	C	0.334	1.017	0.144	2.20E-12	0.324	-0.005	0.010	0.604
rs61796569	1:66476437	T	C	0.27	0.927	0.154	1.50E-09	0.261	-0.008	0.010	0.456
rs61985058	14:60233841	T	C	0.143	1.116	0.194	1.30E-08	0.132	0.013	0.014	0.334
rs62120041	2:9185564	T	C	0.934	1.567	0.274	9.60E-09	0.937	-0.038	0.019	0.049

rs6575005	14:26954078	T	C	0.758	0.934	0.159	4.40E-09	0.764	0.006	0.011	0.582
rs7115226	11:113408518	A	C	0.074	1.594	0.261	1.70E-09	0.072	-0.014	0.019	0.463
rs72804080	2:59358659	G	A	0.15	1.068	0.192	2.90E-08	0.846	0.007	0.013	0.597
rs73219758	8:14279446	G	A	0.708	0.984	0.15	5.60E-11	0.286	-0.007	0.010	0.497
rs7503199	17:8134275	C	T	0.734	0.885	0.154	1.00E-08	0.263	0.006	0.011	0.611
rs75539574	2:58871658	C	A	0.086	2.175	0.244	6.90E-19	0.912	0.012	0.017	0.483
rs7556815	2:114085785	A	G	0.219	2.443	0.164	1.30E-49	0.227	-0.005	0.011	0.642
rs7616632	3:137031237	T	G	0.522	0.792	0.136	4.30E-09	0.526	-0.001	0.009	0.881
rs7644809	3:107564459	T	C	0.422	0.784	0.138	1.60E-08	0.426	0.004	0.009	0.673
rs7806045	7:132610266	T	C	0.755	0.887	0.158	1.40E-08	0.753	-0.019	0.011	0.079
rs7915425	10:125016501	T	C	0.175	1.144	0.179	2.00E-10	0.187	0.013	0.012	0.297
rs7951019	11:118358027	G	T	0.032	2.213	0.391	1.20E-08	0.970	-0.044	0.032	0.166
rs80193650	6:33464363	G	A	0.162	1.01	0.184	4.10E-08	0.840	0.001	0.013	0.940
rs8038326	15:47989799	A	G	0.727	0.955	0.152	2.80E-10	0.717	0.002	0.010	0.834
rs8050478	16:56120461	G	A	0.5	0.96	0.136	1.70E-12	0.490	0.005	0.009	0.563
rs915416	1:34731984	C	G	0.29	1.156	0.15	9.90E-15	0.292	0.008	0.010	0.437
rs9345234	6:93162639	C	A	0.578	0.781	0.138	1.80E-08	0.434	-0.006	0.010	0.573
rs9382445	6:54937974	T	C	0.623	0.872	0.14	4.80E-10	0.637	-0.006	0.010	0.560
rs9903973	17:50571227	C	T	0.467	0.766	0.136	2.60E-08	0.538	0.032	0.009	0.000
rs9940646	16:53800629	C	G	0.578	1.017	0.137	1.20E-13	0.566	-0.009	0.009	0.325

Note1: EA, effect allele; OA, other allele; EAF, effect allele frequency.

Note2: ICH data not included.

Table S6. Harmonised Datasets for the MR Analyses of sleep exposures on any ischemic Stroke

exposure	SNP	Chr:Pos	EA	OA	Association with exposures				Any ischemic Stroke			
					EAF	Beta	Se	Pval	EAF	Beta	Se	Pval
Short sleep	rs2863957	2:114089551	A	C	0.782	0.054	0.007	2.60E-18	0.226	-0.003	0.012	0.822
	rs13107325	4:103188709	T	C	0.075	0.075	0.011	2.50E-13	0.075	-0.007	0.022	0.761
	rs1229762	7:114218582	T	C	0.665	0.037	0.006	1.10E-12	0.656	-0.001	0.011	0.961
	rs1380703	2:57941287	A	G	0.384	0.035	0.006	1.60E-11	0.619	-0.004	0.011	0.709
	rs12963463	18:53099093	T	C	0.299	0.029	0.006	1.90E-11	0.705	0.009	0.011	0.436
	rs75539574	2:58871658	A	C	0.915	0.045	0.011	8.40E-11	0.912	0.022	0.018	0.227
	rs17388803	15:48027204	A	C	0.106	0.053	0.010	6.50E-10	0.878	-0.032	0.016	0.044
	rs4585442	5:135508381	A	G	0.311	0.031	0.006	8.10E-10	0.697	-0.003	0.011	0.747
	rs1607227	11:28808617	T	G	0.705	0.031	0.007	1.50E-09	0.298	0.005	0.011	0.654
	rs2820313	1:201870221	A	G	0.341	0.031	0.006	2.30E-09	0.672	-0.017	0.011	0.104
	rs17005118	4:82288564	A	G	0.265	0.030	0.007	2.50E-09	0.268	0.012	0.011	0.286
	rs5757675	22:39838892	T	G	0.260	0.034	0.007	2.70E-09	0.722	-0.002	0.011	0.836
	rs12567114	1:98527951	A	G	0.725	0.036	0.007	4.10E-09	0.280	0.006	0.011	0.588
	rs142180737	6:28344731	C	T	0.009	0.154	0.032	4.40E-09	NA	NA	NA	NA
	rs2186122	1:66470206	A	T	0.562	0.024	0.006	4.80E-09	0.437	-0.016	0.010	0.111
	rs11763750	7:2080114	A	G	0.814	0.035	0.008	5.10E-09	0.200	-0.012	0.013	0.360
	rs12518468	5:7249696	T	C	0.328	0.031	0.006	8.50E-09	0.677	-0.001	0.011	0.953
	rs9367621	6:55040290	A	T	0.431	0.024	0.006	1.60E-08	0.582	-0.003	0.010	0.789

	rs3776864	5:102327868	A	C	0.667	0.031	0.006	1.70E-08	0.696	-0.003	0.011	0.814
	rs60882754	8:52886619	A	T	0.939	0.055	0.012	1.80E-08	0.946	0.009	0.023	0.693
	rs59779556	16:56227965	T	G	0.554	0.025	0.006	2.00E-08	0.538	-0.001	0.010	0.890
	rs2014830	3:50172397	T	C	0.698	0.030	0.006	2.70E-08	0.316	-0.012	0.011	0.274
	rs205024	17:11227352	T	C	0.617	0.031	0.006	2.70E-08	0.395	-0.010	0.010	0.316
	rs12661667	6:41792545	T	C	0.263	0.028	0.007	2.80E-08	0.253	-0.015	0.011	0.200
	rs7939345	11:47980568	T	G	0.208	0.035	0.007	4.00E-08	0.209	0.002	0.013	0.878
	rs9321171	6:129848635	T	C	0.540	0.031	0.006	4.20E-08	0.454	-0.001	0.010	0.891
	rs7524118	1:34736052	T	C	0.708	0.030	0.006	4.90E-08	0.296	0.015	0.011	0.173
Long sleep	rs6737318	2:114083120	G	A	0.222	0.076	0.011	3.40E-13	0.775	0.001	0.012	0.917
	rs75458655	11:118115331	T	C	0.023	0.185	0.029	5.40E-12	0.023	0.005	0.042	0.909
	rs17817288	16:53807764	A	G	0.518	0.039	0.009	8.90E-09	0.506	-0.011	0.010	0.271
	rs549961083	5:58184093	T	C	0.001	0.534	0.117	9.60E-09	NA	NA	NA	NA
	rs3751046	11:122828342	G	A	0.147	0.070	0.013	2.00E-08	0.852	0.002	0.014	0.901
	rs7534398	1:7767464	A	T	0.201	0.047	0.012	2.10E-08	0.196	0.012	0.013	0.332
	rs10899257	11:76415209	A	G	0.144	0.068	0.013	4.60E-08	0.145	-0.008	0.014	0.582
	rs17688916	17:43778680	T	A	0.796	0.071	0.012	1.10E-11	0.207	0.011	0.017	0.509
Sleep duration	rs10173260	2:210377845	C	T	0.606	0.77	0.139	2.90E-08	0.389	-0.002	0.010	0.833
	rs10421649	19:9942262	A	T	0.557	0.798	0.138	6.90E-09	0.515	0.009	0.011	0.379
	rs10483350	14:29816155	G	A	0.195	1.042	0.172	1.50E-09	0.801	-0.030	0.013	0.020
	rs1057703	11:122830251	G	T	0.147	1.164	0.192	1.10E-09	0.852	0.001	0.014	0.944
	rs10761674	10:64618340	C	T	0.477	0.74	0.136	4.20E-08	0.519	0.000	0.010	0.965
	rs10973207	9:37100525	T	G	0.158	1.226	0.187	6.00E-11	0.161	0.007	0.014	0.635
	rs11190970	10:103128332	G	A	0.799	0.923	0.169	4.60E-08	0.208	0.000	0.013	0.986
	rs112230981	3:55879269	A	G	0.95	1.892	0.314	2.20E-09	0.954	-0.055	0.026	0.032

rs113113059	6:43160375	T	C	0.78	0.968	0.164	8.40E-09	0.791	-0.005	0.012	0.684
rs114614603	6:28584775	A	G	0.72	1.015	0.151	2.30E-11	0.746	0.005	0.016	0.774
rs11567976	5:137654218	T	C	0.571	0.768	0.137	2.10E-08	0.550	-0.012	0.010	0.225
rs11602180	11:48162453	C	T	0.837	1.095	0.184	2.30E-09	0.168	0.001	0.014	0.948
rs11614986	12:110007939	A	G	0.821	0.983	0.177	2.70E-08	0.822	0.001	0.013	0.960
rs11621908	14:78495761	C	T	0.917	1.446	0.25	5.60E-09	0.085	-0.004	0.019	0.839
rs11643715	16:23909538	G	C	0.291	0.834	0.15	3.20E-08	0.712	-0.004	0.011	0.689
rs117527039	17:44083402	G	A	0.774	0.994	0.163	1.00E-09	0.225	0.009	0.016	0.559
rs11885663	2:166944004	T	C	0.248	0.973	0.157	8.60E-10	0.257	0.018	0.011	0.111
rs12246842	10:21830580	A	G	0.46	0.804	0.136	3.90E-09	0.451	0.009	0.010	0.369
rs12567114	1:98527951	A	G	0.276	0.89	0.152	4.30E-09	0.280	0.006	0.011	0.588
rs12607679	18:53059748	T	C	0.738	1.208	0.156	8.30E-15	0.749	0.010	0.012	0.386
rs12611523	2:139195328	A	G	0.545	0.758	0.137	3.10E-08	0.552	0.005	0.010	0.606
rs1263056	11:116576415	A	G	0.519	0.768	0.137	2.00E-08	0.508	-0.005	0.010	0.639
rs12791153	11:80685181	T	A	0.081	1.413	0.253	1.90E-08	0.915	0.005	0.019	0.816
rs13088093	3:135838598	G	T	0.336	0.976	0.144	7.00E-12	0.667	0.021	0.011	0.050
rs13109404	4:102896591	T	G	0.928	1.872	0.264	1.40E-12	0.936	-0.002	0.023	0.937
rs151014368	5:176751059	A	G	0.206	0.966	0.169	9.10E-09	0.230	-0.007	0.012	0.563
rs1517572	11:28829882	C	A	0.581	0.879	0.138	1.50E-10	0.415	0.002	0.010	0.813
rs1553132	11:88297740	G	A	0.258	0.87	0.155	2.50E-08	0.741	0.014	0.011	0.230
rs17427571	4:82254908	A	G	0.684	0.83	0.146	1.30E-08	0.673	0.006	0.011	0.578
rs174560	11:61581764	C	T	0.314	0.815	0.146	2.80E-08	0.691	0.028	0.011	0.013
rs17732997	3:70470834	C	G	0.569	0.776	0.137	1.20E-08	0.581	-0.001	0.010	0.942
rs1776776	9:140497072	T	C	0.874	1.198	0.205	4.90E-09	0.860	-0.009	0.015	0.527
rs180769	5:135615615	T	C	0.425	0.763	0.138	2.30E-08	0.436	0.009	0.011	0.394
rs1939455	11:101520886	G	T	0.879	1.226	0.214	1.20E-08	0.116	-0.019	0.017	0.262

rs205024	17:11227352	T	C	0.384	0.83	0.14	3.90E-09	0.395	-0.010	0.010	0.316
rs2072727	20:43538733	T	C	0.436	0.795	0.137	7.90E-09	0.430	0.000	0.010	0.989
rs2079070	7:114126432	C	G	0.265	1.053	0.154	7.50E-12	0.271	-0.006	0.012	0.618
rs2139261	17:21313223	G	C	0.749	1.122	0.174	8.50E-11	NA	NA	NA	NA
rs2192528	4:18327896	A	G	0.48	0.802	0.136	2.70E-09	0.474	-0.003	0.010	0.743
rs2231265	6:89790201	G	A	0.772	0.897	0.162	2.70E-08	0.228	-0.009	0.012	0.441
rs269054	1:57864304	A	T	0.422	0.819	0.138	2.10E-09	0.411	-0.001	0.011	0.960
rs3095508	16:6550400	C	A	0.594	0.921	0.138	3.10E-11	0.384	-0.012	0.010	0.246
rs330088	8:9149746	C	T	0.547	0.868	0.137	2.70E-10	0.469	0.008	0.011	0.453
rs34354917	12:38764559	C	A	0.71	0.825	0.15	3.90E-08	0.287	0.009	0.012	0.469
rs34731055	7:2106928	T	C	0.181	1.168	0.177	3.70E-11	0.200	-0.006	0.013	0.660
rs35531607	4:92533225	C	T	0.474	0.77	0.136	1.50E-08	0.537	0.005	0.010	0.592
rs365663	5:1428883	A	G	0.546	0.878	0.137	1.00E-10	0.542	0.000	0.010	0.997
rs374153	2:40382712	C	T	0.158	1.057	0.186	9.10E-09	0.847	-0.004	0.015	0.769
rs4128364	2:147612734	C	T	0.339	0.876	0.143	1.40E-09	0.652	0.034	0.011	0.001
rs4538155	2:157040773	T	C	0.647	0.779	0.142	3.60E-08	0.653	0.021	0.010	0.046
rs4592416	11:43800474	G	A	0.464	0.881	0.136	9.30E-11	0.543	0.028	0.010	0.004
rs460692	5:3126584	C	T	0.137	1.263	0.2	3.60E-10	0.851	-0.006	0.015	0.666
rs4767550	12:117951150	G	A	0.414	0.858	0.139	6.30E-10	0.584	-0.004	0.010	0.697
rs55658675	14:65554638	C	T	0.645	0.788	0.142	2.00E-08	0.346	-0.004	0.011	0.709
rs56372231	5:102321905	T	C	0.334	1.017	0.144	2.20E-12	0.324	0.000	0.011	0.975
rs61796569	1:66476437	T	C	0.27	0.927	0.154	1.50E-09	0.262	-0.001	0.011	0.905
rs61985058	14:60233841	T	C	0.143	1.116	0.194	1.30E-08	0.133	0.014	0.015	0.347
rs62120041	2:9185564	T	C	0.934	1.567	0.274	9.60E-09	0.937	-0.021	0.021	0.325
rs6575005	14:26954078	T	C	0.758	0.934	0.159	4.40E-09	0.763	0.013	0.013	0.326
rs7115226	11:113408518	A	C	0.074	1.594	0.261	1.70E-09	0.071	-0.025	0.020	0.222

rs72804080	2:59358659	G	A	0.15	1.068	0.192	2.90E-08	0.846	0.001	0.014	0.920
rs73219758	8:14279446	G	A	0.708	0.984	0.15	5.60E-11	0.286	-0.001	0.011	0.898
rs7503199	17:8134275	C	T	0.734	0.885	0.154	1.00E-08	0.263	0.002	0.012	0.857
rs75539574	2:58871658	C	A	0.086	2.175	0.244	6.90E-19	0.912	0.022	0.018	0.227
rs7556815	2:114085785	A	G	0.219	2.443	0.164	1.30E-49	0.227	-0.001	0.012	0.935
rs7616632	3:137031237	T	G	0.522	0.792	0.136	4.30E-09	0.527	-0.004	0.010	0.689
rs7644809	3:107564459	T	C	0.422	0.784	0.138	1.60E-08	0.425	0.001	0.010	0.954
rs7806045	7:132610266	T	C	0.755	0.887	0.158	1.40E-08	0.753	-0.014	0.012	0.240
rs7915425	10:125016501	T	C	0.175	1.144	0.179	2.00E-10	0.186	0.028	0.013	0.034
rs7951019	11:118358027	G	T	0.032	2.213	0.391	1.20E-08	0.970	-0.035	0.035	0.312
rs80193650	6:33464363	G	A	0.162	1.01	0.184	4.10E-08	0.840	-0.005	0.014	0.705
rs8038326	15:47989799	A	G	0.727	0.955	0.152	2.80E-10	0.717	-0.006	0.011	0.610
rs8050478	16:56120461	G	A	0.5	0.96	0.136	1.70E-12	0.491	0.000	0.010	0.993
rs915416	1:34731984	C	G	0.29	1.156	0.15	9.90E-15	0.293	0.014	0.011	0.213
rs9345234	6:93162639	C	A	0.578	0.781	0.138	1.80E-08	0.434	-0.006	0.010	0.567
rs9382445	6:54937974	T	C	0.623	0.872	0.14	4.80E-10	0.640	0.006	0.011	0.601
rs9903973	17:50571227	C	T	0.467	0.766	0.136	2.60E-08	0.539	0.031	0.010	0.002
rs9940646	16:53800629	C	G	0.578	1.017	0.137	1.20E-13	0.566	-0.011	0.010	0.268

Note1: EA, effect allele; OA, other allele; EAF, effect allele frequency.

Note2: ICH data not included.

Table S7. Harmonised Datasets for the MR Analyses of sleep exposures on large artery Stroke

exposure	SNP	Chr:Pos	EA	OA	Association with exposures				Large artery stroke			
					EAF	Beta	Se	Pval	EAF	Beta	Se	Pval
Short sleep	rs2863957	2:114089551	A	C	0.782	0.054	0.007	2.60E-18	0.225	-0.004	0.030	0.888
	rs13107325	4:103188709	T	C	0.075	0.075	0.011	2.50E-13	0.080	0.021	0.053	0.697
	rs1229762	7:114218582	T	C	0.665	0.037	0.006	1.10E-12	0.651	0.028	0.026	0.290
	rs1380703	2:57941287	A	G	0.384	0.035	0.006	1.60E-11	0.621	0.013	0.026	0.610
	rs12963463	18:53099093	T	C	0.299	0.029	0.006	1.90E-11	0.704	-0.013	0.027	0.637
	rs75539574	2:58871658	A	C	0.915	0.045	0.011	8.40E-11	0.912	0.075	0.047	0.107
	rs17388803	15:48027204	A	C	0.106	0.053	0.010	6.50E-10	0.877	-0.013	0.039	0.732
	rs4585442	5:135508381	A	G	0.311	0.031	0.006	8.10E-10	0.697	0.028	0.027	0.316
	rs1607227	11:28808617	T	G	0.705	0.031	0.007	1.50E-09	0.294	0.022	0.028	0.424
	rs2820313	1:201870221	A	G	0.341	0.031	0.006	2.30E-09	0.673	-0.019	0.026	0.476
	rs17005118	4:82288564	A	G	0.265	0.030	0.007	2.50E-09	0.265	0.028	0.028	0.325
	rs5757675	22:39838892	T	G	0.260	0.034	0.007	2.70E-09	0.714	0.012	0.028	0.650
	rs12567114	1:98527951	A	G	0.725	0.036	0.007	4.10E-09	0.281	-0.013	0.028	0.649
	rs142180737	6:28344731	C	T	0.009	0.154	0.032	4.40E-09	0.986	-0.174	0.255	0.494
	rs2186122	1:66470206	A	T	0.562	0.024	0.006	4.80E-09	0.438	-0.012	0.025	0.621
	rs11763750	7:2080114	A	G	0.814	0.035	0.008	5.10E-09	0.201	-0.025	0.031	0.433
	rs12518468	5:7249696	T	C	0.328	0.031	0.006	8.50E-09	0.675	-0.004	0.027	0.870
	rs9367621	6:55040290	A	T	0.431	0.024	0.006	1.60E-08	0.579	-0.024	0.025	0.345

	rs3776864	5:102327868	A	C	0.667	0.031	0.006	1.70E-08	0.695	0.028	0.027	0.309
	rs60882754	8:52886619	A	T	0.939	0.055	0.012	1.80E-08	0.946	0.072	0.059	0.222
	rs59779556	16:56227965	T	G	0.554	0.025	0.006	2.00E-08	0.539	-0.012	0.025	0.645
	rs2014830	3:50172397	T	C	0.698	0.030	0.006	2.70E-08	0.316	-0.024	0.027	0.385
	rs205024	17:11227352	T	C	0.617	0.031	0.006	2.70E-08	0.399	-0.068	0.026	0.009
	rs12661667	6:41792545	T	C	0.263	0.028	0.007	2.80E-08	0.254	-0.015	0.028	0.582
	rs7939345	11:47980568	T	G	0.208	0.035	0.007	4.00E-08	0.213	0.046	0.031	0.131
	rs9321171	6:129848635	T	C	0.540	0.031	0.006	4.20E-08	0.452	-0.026	0.025	0.305
	rs7524118	1:34736052	T	C	0.708	0.030	0.006	4.90E-08	0.297	0.070	0.027	0.011
Long sleep	rs6737318	2:114083120	G	A	0.222	0.076	0.011	3.40E-13	0.777	-0.001	0.030	0.968
	rs75458655	11:118115331	T	C	0.023	0.185	0.029	5.40E-12	0.023	0.167	0.105	0.112
	rs17817288	16:53807764	A	G	0.518	0.039	0.009	8.90E-09	0.502	-0.020	0.025	0.419
	rs549961083	5:58184093	T	C	0.001	0.534	0.117	9.60E-09	NA	NA	NA	NA
	rs3751046	11:122828342	G	A	0.147	0.070	0.013	2.00E-08	0.852	-0.030	0.035	0.396
	rs7534398	1:7767464	A	T	0.201	0.047	0.012	2.10E-08	0.198	0.029	0.031	0.346
	rs10899257	11:76415209	A	G	0.144	0.068	0.013	4.60E-08	0.147	0.004	0.035	0.913
	rs17688916	17:43778680	T	A	0.796	0.071	0.012	1.10E-11	0.214	-0.017	0.040	0.670
Sleep duration	rs10173260	2:210377845	C	T	0.606	0.77	0.139	2.90E-08	0.389	-0.031	0.025	0.215
	rs10421649	19:9942262	A	T	0.557	0.798	0.138	6.90E-09	0.506	-0.002	0.027	0.930
	rs10483350	14:29816155	G	A	0.195	1.042	0.172	1.50E-09	0.801	-0.010	0.031	0.750
	rs1057703	11:122830251	G	T	0.147	1.164	0.192	1.10E-09	0.852	-0.030	0.036	0.400
	rs10761674	10:64618340	C	T	0.477	0.74	0.136	4.20E-08	0.516	0.000	0.024	0.988
	rs10973207	9:37100525	T	G	0.158	1.226	0.187	6.00E-11	0.162	0.031	0.034	0.368
	rs11190970	10:103128332	G	A	0.799	0.923	0.169	4.60E-08	0.210	-0.004	0.031	0.907
	rs112230981	3:55879269	A	G	0.95	1.892	0.314	2.20E-09	0.954	-0.025	0.068	0.708

rs113113059	6:43160375	T	C	0.78	0.968	0.164	8.40E-09	0.791	-0.014	0.030	0.642
rs114614603	6:28584775	A	G	0.72	1.015	0.151	2.30E-11	0.747	-0.065	0.036	0.075
rs11567976	5:137654218	T	C	0.571	0.768	0.137	2.10E-08	0.545	-0.060	0.025	0.015
rs11602180	11:48162453	C	T	0.837	1.095	0.184	2.30E-09	0.170	0.074	0.034	0.027
rs11614986	12:110007939	A	G	0.821	0.983	0.177	2.70E-08	0.822	-0.011	0.032	0.728
rs11621908	14:78495761	C	T	0.917	1.446	0.25	5.60E-09	0.090	-0.022	0.046	0.631
rs11643715	16:23909538	G	C	0.291	0.834	0.15	3.20E-08	0.712	-0.007	0.027	0.805
rs117527039	17:44083402	G	A	0.774	0.994	0.163	1.00E-09	0.233	-0.012	0.037	0.741
rs11885663	2:166944004	T	C	0.248	0.973	0.157	8.60E-10	0.259	0.048	0.028	0.084
rs12246842	10:21830580	A	G	0.46	0.804	0.136	3.90E-09	0.446	0.005	0.025	0.853
rs12567114	1:98527951	A	G	0.276	0.89	0.152	4.30E-09	0.281	-0.013	0.028	0.649
rs12607679	18:53059748	T	C	0.738	1.208	0.156	8.30E-15	0.747	-0.002	0.028	0.950
rs12611523	2:139195328	A	G	0.545	0.758	0.137	3.10E-08	0.547	0.045	0.025	0.076
rs1263056	11:116576415	A	G	0.519	0.768	0.137	2.00E-08	0.502	-0.021	0.026	0.422
rs12791153	11:80685181	T	A	0.081	1.413	0.253	1.90E-08	0.916	0.062	0.050	0.211
rs13088093	3:135838598	G	T	0.336	0.976	0.144	7.00E-12	0.668	0.030	0.026	0.248
rs13109404	4:102896591	T	G	0.928	1.872	0.264	1.40E-12	0.932	-0.054	0.055	0.331
rs151014368	5:176751059	A	G	0.206	0.966	0.169	9.10E-09	0.228	-0.034	0.030	0.263
rs1517572	11:28829882	C	A	0.581	0.879	0.138	1.50E-10	0.420	0.005	0.025	0.832
rs1553132	11:88297740	G	A	0.258	0.87	0.155	2.50E-08	0.744	0.004	0.028	0.902
rs17427571	4:82254908	A	G	0.684	0.83	0.146	1.30E-08	0.675	-0.013	0.026	0.619
rs174560	11:61581764	C	T	0.314	0.815	0.146	2.80E-08	0.697	0.079	0.028	0.005
rs17732997	3:70470834	C	G	0.569	0.776	0.137	1.20E-08	0.581	0.002	0.025	0.933
rs1776776	9:140497072	T	C	0.874	1.198	0.205	4.90E-09	0.860	-0.019	0.037	0.607
rs180769	5:135615615	T	C	0.425	0.763	0.138	2.30E-08	0.438	0.026	0.026	0.316
rs1939455	11:101520886	G	T	0.879	1.226	0.214	1.20E-08	0.117	-0.035	0.041	0.398

rs205024	17:11227352	T	C	0.384	0.83	0.14	3.90E-09	0.399	-0.068	0.026	0.009
rs2072727	20:43538733	T	C	0.436	0.795	0.137	7.90E-09	0.427	-0.044	0.025	0.078
rs2079070	7:114126432	C	G	0.265	1.053	0.154	7.50E-12	0.274	-0.012	0.028	0.670
rs2139261	17:21313223	G	C	0.749	1.122	0.174	8.50E-11	NA	NA	NA	NA
rs2192528	4:18327896	A	G	0.48	0.802	0.136	2.70E-09	0.471	-0.022	0.025	0.385
rs2231265	6:89790201	G	A	0.772	0.897	0.162	2.70E-08	0.231	-0.024	0.029	0.423
rs269054	1:57864304	A	T	0.422	0.819	0.138	2.10E-09	0.406	-0.018	0.026	0.490
rs3095508	16:6550400	C	A	0.594	0.921	0.138	3.10E-11	0.381	-0.033	0.025	0.192
rs330088	8:9149746	C	T	0.547	0.868	0.137	2.70E-10	0.472	-0.018	0.026	0.493
rs34354917	12:38764559	C	A	0.71	0.825	0.15	3.90E-08	0.288	0.003	0.030	0.917
rs34731055	7:2106928	T	C	0.181	1.168	0.177	3.70E-11	0.201	-0.018	0.032	0.570
rs35531607	4:92533225	C	T	0.474	0.77	0.136	1.50E-08	0.533	-0.019	0.025	0.431
rs365663	5:1428883	A	G	0.546	0.878	0.137	1.00E-10	0.539	-0.019	0.025	0.440
rs374153	2:40382712	C	T	0.158	1.057	0.186	9.10E-09	0.849	-0.001	0.037	0.977
rs4128364	2:147612734	C	T	0.339	0.876	0.143	1.40E-09	0.652	0.001	0.026	0.967
rs4538155	2:157040773	T	C	0.647	0.779	0.142	3.60E-08	0.652	-0.019	0.026	0.459
rs4592416	11:43800474	G	A	0.464	0.881	0.136	9.30E-11	0.543	0.063	0.025	0.011
rs460692	5:3126584	C	T	0.137	1.263	0.2	3.60E-10	0.854	0.011	0.037	0.778
rs4767550	12:117951150	G	A	0.414	0.858	0.139	6.30E-10	0.586	-0.060	0.026	0.018
rs55658675	14:65554638	C	T	0.645	0.788	0.142	2.00E-08	0.343	-0.015	0.026	0.578
rs56372231	5:102321905	T	C	0.334	1.017	0.144	2.20E-12	0.327	-0.019	0.026	0.470
rs61796569	1:66476437	T	C	0.27	0.927	0.154	1.50E-09	0.261	0.006	0.028	0.840
rs61985058	14:60233841	T	C	0.143	1.116	0.194	1.30E-08	0.133	-0.007	0.038	0.862
rs62120041	2:9185564	T	C	0.934	1.567	0.274	9.60E-09	0.936	-0.070	0.052	0.179
rs6575005	14:26954078	T	C	0.758	0.934	0.159	4.40E-09	0.764	0.044	0.030	0.134
rs7115226	11:113408518	A	C	0.074	1.594	0.261	1.70E-09	0.071	-0.046	0.052	0.381

rs72804080	2:59358659	G	A	0.15	1.068	0.192	2.90E-08	0.847	-0.027	0.035	0.446
rs73219758	8:14279446	G	A	0.708	0.984	0.15	5.60E-11	0.287	0.036	0.027	0.179
rs7503199	17:8134275	C	T	0.734	0.885	0.154	1.00E-08	0.266	-0.002	0.028	0.953
rs75539574	2:58871658	C	A	0.086	2.175	0.244	6.90E-19	0.912	0.075	0.047	0.107
rs7556815	2:114085785	A	G	0.219	2.443	0.164	1.30E-49	0.225	-0.003	0.031	0.930
rs7616632	3:137031237	T	G	0.522	0.792	0.136	4.30E-09	0.529	-0.024	0.025	0.325
rs7644809	3:107564459	T	C	0.422	0.784	0.138	1.60E-08	0.427	0.010	0.025	0.705
rs7806045	7:132610266	T	C	0.755	0.887	0.158	1.40E-08	0.754	-0.011	0.029	0.695
rs7915425	10:125016501	T	C	0.175	1.144	0.179	2.00E-10	0.191	0.026	0.032	0.415
rs7951019	11:118358027	G	T	0.032	2.213	0.391	1.20E-08	0.970	0.005	0.094	0.959
rs80193650	6:33464363	G	A	0.162	1.01	0.184	4.10E-08	0.838	0.006	0.034	0.859
rs8038326	15:47989799	A	G	0.727	0.955	0.152	2.80E-10	0.716	0.011	0.027	0.672
rs8050478	16:56120461	G	A	0.5	0.96	0.136	1.70E-12	0.496	-0.004	0.025	0.889
rs915416	1:34731984	C	G	0.29	1.156	0.15	9.90E-15	0.294	0.068	0.027	0.012
rs9345234	6:93162639	C	A	0.578	0.781	0.138	1.80E-08	0.436	-0.047	0.025	0.062
rs9382445	6:54937974	T	C	0.623	0.872	0.14	4.80E-10	0.639	-0.014	0.027	0.613
rs9903973	17:50571227	C	T	0.467	0.766	0.136	2.60E-08	0.538	0.001	0.025	0.964
rs9940646	16:53800629	C	G	0.578	1.017	0.137	1.20E-13	0.564	-0.049	0.025	0.049

Note1: EA, effect allele; OA, other allele; EAF, effect allele frequency.

Note2: ICH data not included.

Table S8. Harmonised Datasets for the MR Analyses of sleep exposures on cardioembolic stroke

exposure	SNP	Chr:Pos	EA	OA	Association with exposures				Cardioembolic stroke			
					EAF	Beta	Se	Pval	EAF	Beta	Se	Pval
Short sleep	rs2863957	2:114089551	A	C	0.782	0.054	0.007	2.60E-18	0.226	0.020	0.023	0.396
	rs13107325	4:103188709	T	C	0.075	0.075	0.011	2.50E-13	0.077	-0.030	0.044	0.492
	rs1229762	7:114218582	T	C	0.665	0.037	0.006	1.10E-12	0.655	0.013	0.020	0.523
	rs1380703	2:57941287	A	G	0.384	0.035	0.006	1.60E-11	0.620	0.012	0.020	0.555
	rs12963463	18:53099093	T	C	0.299	0.029	0.006	1.90E-11	0.706	0.004	0.021	0.869
	rs75539574	2:58871658	A	C	0.915	0.045	0.011	8.40E-11	0.913	0.065	0.036	0.070
	rs17388803	15:48027204	A	C	0.106	0.053	0.010	6.50E-10	0.876	-0.074	0.030	0.013
	rs4585442	5:135508381	A	G	0.311	0.031	0.006	8.10E-10	0.697	-0.022	0.021	0.297
	rs1607227	11:28808617	T	G	0.705	0.031	0.007	1.50E-09	0.297	-0.014	0.022	0.523
	rs2820313	1:201870221	A	G	0.341	0.031	0.006	2.30E-09	0.673	0.004	0.020	0.851
	rs17005118	4:82288564	A	G	0.265	0.030	0.007	2.50E-09	0.268	0.022	0.022	0.304
	rs5757675	22:39838892	T	G	0.260	0.034	0.007	2.70E-09	0.718	-0.046	0.021	0.031
	rs12567114	1:98527951	A	G	0.725	0.036	0.007	4.10E-09	0.281	0.018	0.022	0.421
	rs142180737	6:28344731	C	T	0.009	0.154	0.032	4.40E-09	NA	NA	NA	NA
	rs2186122	1:66470206	A	T	0.562	0.024	0.006	4.80E-09	0.438	-0.023	0.019	0.228
	rs11763750	7:2080114	A	G	0.814	0.035	0.008	5.10E-09	0.201	0.010	0.024	0.697
	rs12518468	5:7249696	T	C	0.328	0.031	0.006	8.50E-09	0.676	-0.016	0.021	0.441
	rs9367621	6:55040290	A	T	0.431	0.024	0.006	1.60E-08	0.583	0.002	0.019	0.921

	rs3776864	5:102327868	A	C	0.667	0.031	0.006	1.70E-08	0.694	-0.027	0.021	0.204
	rs60882754	8:52886619	A	T	0.939	0.055	0.012	1.80E-08	0.947	-0.032	0.045	0.484
	rs59779556	16:56227965	T	G	0.554	0.025	0.006	2.00E-08	0.538	-0.018	0.019	0.354
	rs2014830	3:50172397	T	C	0.698	0.030	0.006	2.70E-08	0.316	0.031	0.021	0.143
	rs205024	17:11227352	T	C	0.617	0.031	0.006	2.70E-08	0.397	-0.007	0.020	0.726
	rs12661667	6:41792545	T	C	0.263	0.028	0.007	2.80E-08	0.253	-0.028	0.022	0.212
	rs7939345	11:47980568	T	G	0.208	0.035	0.007	4.00E-08	0.211	-0.027	0.024	0.278
	rs9321171	6:129848635	T	C	0.540	0.031	0.006	4.20E-08	0.453	0.012	0.019	0.527
	rs7524118	1:34736052	T	C	0.708	0.030	0.006	4.90E-08	0.296	0.032	0.021	0.128
Long sleep	rs6737318	2:114083120	G	A	0.222	0.076	0.011	3.40E-13	0.775	-0.020	0.023	0.397
	rs75458655	11:118115331	T	C	0.023	0.185	0.029	5.40E-12	0.022	0.040	0.086	0.645
	rs17817288	16:53807764	A	G	0.518	0.039	0.009	8.90E-09	0.503	-0.001	0.019	0.944
	rs549961083	5:58184093	T	C	0.001	0.534	0.117	9.60E-09	NA	NA	NA	NA
	rs3751046	11:122828342	G	A	0.147	0.070	0.013	2.00E-08	0.853	-0.054	0.028	0.052
	rs7534398	1:7767464	A	T	0.201	0.047	0.012	2.10E-08	0.196	0.014	0.024	0.560
	rs10899257	11:76415209	A	G	0.144	0.068	0.013	4.60E-08	0.147	0.012	0.028	0.652
	rs17688916	17:43778680	T	A	0.796	0.071	0.012	1.10E-11	0.215	-0.014	0.032	0.666
Sleep duration	rs10173260	2:210377845	C	T	0.606	0.77	0.139	2.90E-08	0.389	0.016	0.019	0.407
	rs10421649	19:9942262	A	T	0.557	0.798	0.138	6.90E-09	0.510	0.021	0.020	0.312
	rs10483350	14:29816155	G	A	0.195	1.042	0.172	1.50E-09	0.803	-0.031	0.025	0.208
	rs1057703	11:122830251	G	T	0.147	1.164	0.192	1.10E-09	0.853	-0.048	0.027	0.081
	rs10761674	10:64618340	C	T	0.477	0.74	0.136	4.20E-08	0.519	0.049	0.019	0.009
	rs10973207	9:37100525	T	G	0.158	1.226	0.187	6.00E-11	0.163	-0.033	0.027	0.214
	rs11190970	10:103128332	G	A	0.799	0.923	0.169	4.60E-08	0.211	-0.033	0.024	0.170
	rs112230981	3:55879269	A	G	0.95	1.892	0.314	2.20E-09	0.953	-0.084	0.055	0.126

rs113113059	6:43160375	T	C	0.78	0.968	0.164	8.40E-09	0.793	0.004	0.023	0.859
rs114614603	6:28584775	A	G	0.72	1.015	0.151	2.30E-11	0.752	0.007	0.029	0.806
rs11567976	5:137654218	T	C	0.571	0.768	0.137	2.10E-08	0.547	-0.007	0.019	0.723
rs11602180	11:48162453	C	T	0.837	1.095	0.184	2.30E-09	0.168	-0.022	0.027	0.405
rs11614986	12:110007939	A	G	0.821	0.983	0.177	2.70E-08	0.823	0.007	0.025	0.774
rs11621908	14:78495761	C	T	0.917	1.446	0.25	5.60E-09	0.088	0.007	0.036	0.843
rs11643715	16:23909538	G	C	0.291	0.834	0.15	3.20E-08	0.714	-0.012	0.021	0.579
rs117527039	17:44083402	G	A	0.774	0.994	0.163	1.00E-09	0.233	-0.022	0.030	0.456
rs11885663	2:166944004	T	C	0.248	0.973	0.157	8.60E-10	0.258	-0.009	0.022	0.674
rs12246842	10:21830580	A	G	0.46	0.804	0.136	3.90E-09	0.447	0.015	0.019	0.441
rs12567114	1:98527951	A	G	0.276	0.89	0.152	4.30E-09	0.281	0.018	0.022	0.421
rs12607679	18:53059748	T	C	0.738	1.208	0.156	8.30E-15	0.750	0.004	0.022	0.861
rs12611523	2:139195328	A	G	0.545	0.758	0.137	3.10E-08	0.551	-0.010	0.019	0.607
rs1263056	11:116576415	A	G	0.519	0.768	0.137	2.00E-08	0.506	0.019	0.020	0.343
rs12791153	11:80685181	T	A	0.081	1.413	0.253	1.90E-08	0.914	-0.031	0.037	0.406
rs13088093	3:135838598	G	T	0.336	0.976	0.144	7.00E-12	0.665	0.011	0.020	0.577
rs13109404	4:102896591	T	G	0.928	1.872	0.264	1.40E-12	0.936	0.045	0.047	0.337
rs151014368	5:176751059	A	G	0.206	0.966	0.169	9.10E-09	0.233	-0.008	0.023	0.732
rs1517572	11:28829882	C	A	0.581	0.879	0.138	1.50E-10	0.417	0.013	0.020	0.504
rs1553132	11:88297740	G	A	0.258	0.87	0.155	2.50E-08	0.741	-0.002	0.022	0.932
rs17427571	4:82254908	A	G	0.684	0.83	0.146	1.30E-08	0.671	-0.007	0.020	0.724
rs174560	11:61581764	C	T	0.314	0.815	0.146	2.80E-08	0.693	0.052	0.022	0.018
rs17732997	3:70470834	C	G	0.569	0.776	0.137	1.20E-08	0.582	0.016	0.019	0.403
rs1776776	9:140497072	T	C	0.874	1.198	0.205	4.90E-09	0.859	0.035	0.030	0.235
rs180769	5:135615615	T	C	0.425	0.763	0.138	2.30E-08	0.437	0.032	0.020	0.115
rs1939455	11:101520886	G	T	0.879	1.226	0.214	1.20E-08	0.117	0.032	0.031	0.306

rs205024	17:11227352	T	C	0.384	0.83	0.14	3.90E-09	0.397	-0.007	0.020	0.726
rs2072727	20:43538733	T	C	0.436	0.795	0.137	7.90E-09	0.427	-0.028	0.019	0.145
rs2079070	7:114126432	C	G	0.265	1.053	0.154	7.50E-12	0.270	-0.022	0.022	0.327
rs2139261	17:21313223	G	C	0.749	1.122	0.174	8.50E-11	NA	NA	NA	NA
rs2192528	4:18327896	A	G	0.48	0.802	0.136	2.70E-09	0.474	-0.012	0.019	0.535
rs2231265	6:89790201	G	A	0.772	0.897	0.162	2.70E-08	0.229	-0.007	0.023	0.761
rs269054	1:57864304	A	T	0.422	0.819	0.138	2.10E-09	0.411	0.026	0.020	0.197
rs3095508	16:6550400	C	A	0.594	0.921	0.138	3.10E-11	0.380	-0.001	0.020	0.976
rs330088	8:9149746	C	T	0.547	0.868	0.137	2.70E-10	0.475	0.040	0.020	0.047
rs34354917	12:38764559	C	A	0.71	0.825	0.15	3.90E-08	0.291	0.055	0.023	0.015
rs34731055	7:2106928	T	C	0.181	1.168	0.177	3.70E-11	0.201	0.022	0.025	0.369
rs35531607	4:92533225	C	T	0.474	0.77	0.136	1.50E-08	0.536	-0.009	0.019	0.655
rs365663	5:1428883	A	G	0.546	0.878	0.137	1.00E-10	0.542	-0.010	0.019	0.625
rs374153	2:40382712	C	T	0.158	1.057	0.186	9.10E-09	0.850	-0.011	0.029	0.710
rs4128364	2:147612734	C	T	0.339	0.876	0.143	1.40E-09	0.650	0.008	0.020	0.684
rs4538155	2:157040773	T	C	0.647	0.779	0.142	3.60E-08	0.653	0.025	0.020	0.211
rs4592416	11:43800474	G	A	0.464	0.881	0.136	9.30E-11	0.542	0.001	0.019	0.954
rs460692	5:3126584	C	T	0.137	1.263	0.2	3.60E-10	0.852	-0.045	0.028	0.115
rs4767550	12:117951150	G	A	0.414	0.858	0.139	6.30E-10	0.585	0.000	0.020	0.997
rs55658675	14:65554638	C	T	0.645	0.788	0.142	2.00E-08	0.346	-0.014	0.020	0.481
rs56372231	5:102321905	T	C	0.334	1.017	0.144	2.20E-12	0.325	0.024	0.020	0.231
rs61796569	1:66476437	T	C	0.27	0.927	0.154	1.50E-09	0.260	0.007	0.022	0.734
rs61985058	14:60233841	T	C	0.143	1.116	0.194	1.30E-08	0.132	0.034	0.033	0.316
rs62120041	2:9185564	T	C	0.934	1.567	0.274	9.60E-09	0.938	0.015	0.042	0.724
rs6575005	14:26954078	T	C	0.758	0.934	0.159	4.40E-09	0.763	0.002	0.023	0.920
rs7115226	11:113408518	A	C	0.074	1.594	0.261	1.70E-09	0.073	-0.050	0.041	0.221

rs72804080	2:59358659	G	A	0.15	1.068	0.192	2.90E-08	0.845	0.005	0.027	0.852
rs73219758	8:14279446	G	A	0.708	0.984	0.15	5.60E-11	0.286	-0.014	0.021	0.502
rs7503199	17:8134275	C	T	0.734	0.885	0.154	1.00E-08	0.264	0.010	0.022	0.642
rs75539574	2:58871658	C	A	0.086	2.175	0.244	6.90E-19	0.913	0.065	0.036	0.070
rs7556815	2:114085785	A	G	0.219	2.443	0.164	1.30E-49	0.227	0.026	0.024	0.266
rs7616632	3:137031237	T	G	0.522	0.792	0.136	4.30E-09	0.527	-0.026	0.019	0.172
rs7644809	3:107564459	T	C	0.422	0.784	0.138	1.60E-08	0.425	0.024	0.019	0.206
rs7806045	7:132610266	T	C	0.755	0.887	0.158	1.40E-08	0.752	-0.012	0.022	0.600
rs7915425	10:125016501	T	C	0.175	1.144	0.179	2.00E-10	0.190	-0.004	0.025	0.877
rs7951019	11:118358027	G	T	0.032	2.213	0.391	1.20E-08	0.971	-0.098	0.074	0.185
rs80193650	6:33464363	G	A	0.162	1.01	0.184	4.10E-08	0.840	0.007	0.027	0.807
rs8038326	15:47989799	A	G	0.727	0.955	0.152	2.80E-10	0.714	-0.002	0.021	0.921
rs8050478	16:56120461	G	A	0.5	0.96	0.136	1.70E-12	0.492	-0.013	0.019	0.495
rs915416	1:34731984	C	G	0.29	1.156	0.15	9.90E-15	0.293	0.039	0.021	0.063
rs9345234	6:93162639	C	A	0.578	0.781	0.138	1.80E-08	0.436	-0.023	0.019	0.240
rs9382445	6:54937974	T	C	0.623	0.872	0.14	4.80E-10	0.642	0.016	0.020	0.441
rs9903973	17:50571227	C	T	0.467	0.766	0.136	2.60E-08	0.538	0.026	0.019	0.165
rs9940646	16:53800629	C	G	0.578	1.017	0.137	1.20E-13	0.564	-0.008	0.019	0.663

Note1: EA, effect allele; OA, other allele; EAF, effect allele frequency.

Note2: ICH data not included.

Table S9. Harmonised Datasets for the MR Analyses of sleep exposures on small vessel stroke

exposure	SNP	Chr:Pos	EA	OA	Association with exposures				Small vessel stroke			
					EAF	Beta	Se	Pval	EAF	Beta	Se	Pval
Short sleep	rs2863957	2:114089551	A	C	0.782	0.054	0.007	2.60E-18	0.224	-0.001	0.028	0.986
	rs13107325	4:103188709	T	C	0.075	0.075	0.011	2.50E-13	0.077	0.023	0.048	0.622
	rs1229762	7:114218582	T	C	0.665	0.037	0.006	1.10E-12	0.656	-0.010	0.024	0.683
	rs1380703	2:57941287	A	G	0.384	0.035	0.006	1.60E-11	0.619	-0.009	0.025	0.702
	rs12963463	18:53099093	T	C	0.299	0.029	0.006	1.90E-11	0.702	-0.012	0.025	0.634
	rs75539574	2:58871658	A	C	0.915	0.045	0.011	8.40E-11	0.911	-0.014	0.042	0.740
	rs17388803	15:48027204	A	C	0.106	0.053	0.010	6.50E-10	0.879	-0.032	0.037	0.378
	rs4585442	5:135508381	A	G	0.311	0.031	0.006	8.10E-10	0.695	-0.013	0.025	0.597
	rs1607227	11:28808617	T	G	0.705	0.031	0.007	1.50E-09	0.295	0.022	0.026	0.398
	rs2820313	1:201870221	A	G	0.341	0.031	0.006	2.30E-09	0.668	-0.058	0.024	0.016
	rs17005118	4:82288564	A	G	0.265	0.030	0.007	2.50E-09	0.267	-0.026	0.026	0.317
	rs5757675	22:39838892	T	G	0.260	0.034	0.007	2.70E-09	0.723	0.027	0.026	0.301
	rs12567114	1:98527951	A	G	0.725	0.036	0.007	4.10E-09	0.280	0.033	0.026	0.208
	rs142180737	6:28344731	C	T	0.009	0.154	0.032	4.40E-09	NA	NA	NA	NA
	rs2186122	1:66470206	A	T	0.562	0.024	0.006	4.80E-09	0.437	0.004	0.023	0.847
	rs11763750	7:2080114	A	G	0.814	0.035	0.008	5.10E-09	0.196	0.001	0.029	0.973
	rs12518468	5:7249696	T	C	0.328	0.031	0.006	8.50E-09	0.675	-0.017	0.025	0.496
	rs9367621	6:55040290	A	T	0.431	0.024	0.006	1.60E-08	0.576	-0.039	0.023	0.093

	rs3776864	5:102327868	A	C	0.667	0.031	0.006	1.70E-08	0.696	0.029	0.025	0.249
	rs60882754	8:52886619	A	T	0.939	0.055	0.012	1.80E-08	0.944	-0.041	0.052	0.426
	rs59779556	16:56227965	T	G	0.554	0.025	0.006	2.00E-08	0.543	0.031	0.023	0.187
	rs2014830	3:50172397	T	C	0.698	0.030	0.006	2.70E-08	0.313	-0.010	0.025	0.705
	rs205024	17:11227352	T	C	0.617	0.031	0.006	2.70E-08	0.394	0.005	0.024	0.851
	rs12661667	6:41792545	T	C	0.263	0.028	0.007	2.80E-08	0.254	-0.002	0.026	0.933
	rs7939345	11:47980568	T	G	0.208	0.035	0.007	4.00E-08	0.212	-0.052	0.029	0.074
	rs9321171	6:129848635	T	C	0.540	0.031	0.006	4.20E-08	0.456	0.015	0.024	0.521
	rs7524118	1:34736052	T	C	0.708	0.030	0.006	4.90E-08	0.296	0.029	0.026	0.262
	rs6737318	2:114083120	G	A	0.222	0.076	0.011	3.40E-13	0.778	-0.002	0.028	0.958
	rs75458655	11:118115331	T	C	0.023	0.185	0.029	5.40E-12	0.024	-0.022	0.096	0.821
	rs17817288	16:53807764	A	G	0.518	0.039	0.009	8.90E-09	0.507	-0.014	0.023	0.540
Long sleep	rs549961083	5:58184093	T	C	0.001	0.534	0.117	9.60E-09	NA	NA	NA	NA
	rs3751046	11:122828342	G	A	0.147	0.070	0.013	2.00E-08	0.854	-0.017	0.033	0.602
	rs7534398	1:7767464	A	T	0.201	0.047	0.012	2.10E-08	0.195	-0.012	0.029	0.689
	rs10899257	11:76415209	A	G	0.144	0.068	0.013	4.60E-08	0.147	0.036	0.033	0.278
	rs17688916	17:43778680	T	A	0.796	0.071	0.012	1.10E-11	0.213	0.093	0.034	0.006
	rs10173260	2:210377845	C	T	0.606	0.77	0.139	2.90E-08	0.389	0.031	0.024	0.191
	rs10421649	19:9942262	A	T	0.557	0.798	0.138	6.90E-09	0.505	-0.006	0.025	0.805
	rs10483350	14:29816155	G	A	0.195	1.042	0.172	1.50E-09	0.801	-0.042	0.029	0.143
Sleep duration	rs1057703	11:122830251	G	T	0.147	1.164	0.192	1.10E-09	0.853	-0.024	0.033	0.482
	rs10761674	10:64618340	C	T	0.477	0.74	0.136	4.20E-08	0.519	-0.017	0.023	0.447
	rs10973207	9:37100525	T	G	0.158	1.226	0.187	6.00E-11	0.158	0.071	0.032	0.025
	rs11190970	10:103128332	G	A	0.799	0.923	0.169	4.60E-08	0.208	0.036	0.028	0.201
	rs112230981	3:55879269	A	G	0.95	1.892	0.314	2.20E-09	0.953	-0.070	0.060	0.245

rs113113059	6:43160375	T	C	0.78	0.968	0.164	8.40E-09	0.786	-0.043	0.028	0.120
rs114614603	6:28584775	A	G	0.72	1.015	0.151	2.30E-11	0.742	0.047	0.032	0.139
rs11567976	5:137654218	T	C	0.571	0.768	0.137	2.10E-08	0.551	-0.033	0.023	0.152
rs11602180	11:48162453	C	T	0.837	1.095	0.184	2.30E-09	0.168	-0.024	0.032	0.455
rs11614986	12:110007939	A	G	0.821	0.983	0.177	2.70E-08	0.822	0.011	0.030	0.714
rs11621908	14:78495761	C	T	0.917	1.446	0.25	5.60E-09	0.087	-0.026	0.043	0.555
rs11643715	16:23909538	G	C	0.291	0.834	0.15	3.20E-08	0.711	-0.065	0.026	0.011
rs117527039	17:44083402	G	A	0.774	0.994	0.163	1.00E-09	0.232	0.097	0.032	0.002
rs11885663	2:166944004	T	C	0.248	0.973	0.157	8.60E-10	0.256	0.004	0.026	0.884
rs12246842	10:21830580	A	G	0.46	0.804	0.136	3.90E-09	0.449	0.007	0.023	0.771
rs12567114	1:98527951	A	G	0.276	0.89	0.152	4.30E-09	0.280	0.033	0.026	0.208
rs12607679	18:53059748	T	C	0.738	1.208	0.156	8.30E-15	0.745	-0.013	0.026	0.625
rs12611523	2:139195328	A	G	0.545	0.758	0.137	3.10E-08	0.547	-0.006	0.023	0.798
rs1263056	11:116576415	A	G	0.519	0.768	0.137	2.00E-08	0.510	-0.025	0.024	0.291
rs12791153	11:80685181	T	A	0.081	1.413	0.253	1.90E-08	0.917	-0.015	0.047	0.757
rs13088093	3:135838598	G	T	0.336	0.976	0.144	7.00E-12	0.666	0.038	0.024	0.115
rs13109404	4:102896591	T	G	0.928	1.872	0.264	1.40E-12	0.933	-0.019	0.052	0.712
rs151014368	5:176751059	A	G	0.206	0.966	0.169	9.10E-09	0.227	-0.005	0.028	0.851
rs1517572	11:28829882	C	A	0.581	0.879	0.138	1.50E-10	0.421	-0.013	0.024	0.579
rs1553132	11:88297740	G	A	0.258	0.87	0.155	2.50E-08	0.742	0.025	0.026	0.349
rs17427571	4:82254908	A	G	0.684	0.83	0.146	1.30E-08	0.676	0.035	0.024	0.153
rs174560	11:61581764	C	T	0.314	0.815	0.146	2.80E-08	0.691	-0.023	0.026	0.364
rs17732997	3:70470834	C	G	0.569	0.776	0.137	1.20E-08	0.579	0.013	0.023	0.573
rs1776776	9:140497072	T	C	0.874	1.198	0.205	4.90E-09	0.861	-0.009	0.035	0.788
rs180769	5:135615615	T	C	0.425	0.763	0.138	2.30E-08	0.437	0.001	0.024	0.983
rs1939455	11:101520886	G	T	0.879	1.226	0.214	1.20E-08	0.117	0.001	0.038	0.983

rs205024	17:11227352	T	C	0.384	0.83	0.14	3.90E-09	0.394	0.005	0.024	0.851
rs2072727	20:43538733	T	C	0.436	0.795	0.137	7.90E-09	0.431	-0.026	0.023	0.253
rs2079070	7:114126432	C	G	0.265	1.053	0.154	7.50E-12	0.271	-0.020	0.026	0.456
rs2139261	17:21313223	G	C	0.749	1.122	0.174	8.50E-11	NA	NA	NA	NA
rs2192528	4:18327896	A	G	0.48	0.802	0.136	2.70E-09	0.473	0.005	0.023	0.843
rs2231265	6:89790201	G	A	0.772	0.897	0.162	2.70E-08	0.229	0.005	0.027	0.854
rs269054	1:57864304	A	T	0.422	0.819	0.138	2.10E-09	0.402	0.025	0.024	0.297
rs3095508	16:6550400	C	A	0.594	0.921	0.138	3.10E-11	0.384	0.012	0.025	0.626
rs330088	8:9149746	C	T	0.547	0.868	0.137	2.70E-10	0.469	0.018	0.024	0.457
rs34354917	12:38764559	C	A	0.71	0.825	0.15	3.90E-08	0.285	-0.016	0.027	0.571
rs34731055	7:2106928	T	C	0.181	1.168	0.177	3.70E-11	0.197	-0.004	0.029	0.903
rs35531607	4:92533225	C	T	0.474	0.77	0.136	1.50E-08	0.535	-0.044	0.023	0.057
rs365663	5:1428883	A	G	0.546	0.878	0.137	1.00E-10	0.542	0.029	0.024	0.222
rs374153	2:40382712	C	T	0.158	1.057	0.186	9.10E-09	0.846	0.020	0.033	0.545
rs4128364	2:147612734	C	T	0.339	0.876	0.143	1.40E-09	0.652	0.043	0.024	0.078
rs4538155	2:157040773	T	C	0.647	0.779	0.142	3.60E-08	0.652	0.029	0.024	0.222
rs4592416	11:43800474	G	A	0.464	0.881	0.136	9.30E-11	0.542	0.030	0.023	0.191
rs460692	5:3126584	C	T	0.137	1.263	0.2	3.60E-10	0.855	-0.003	0.035	0.937
rs4767550	12:117951150	G	A	0.414	0.858	0.139	6.30E-10	0.585	0.018	0.024	0.447
rs55658675	14:65554638	C	T	0.645	0.788	0.142	2.00E-08	0.345	0.026	0.024	0.277
rs56372231	5:102321905	T	C	0.334	1.017	0.144	2.20E-12	0.327	-0.020	0.024	0.406
rs61796569	1:66476437	T	C	0.27	0.927	0.154	1.50E-09	0.262	0.019	0.026	0.457
rs61985058	14:60233841	T	C	0.143	1.116	0.194	1.30E-08	0.134	-0.023	0.036	0.522
rs62120041	2:9185564	T	C	0.934	1.567	0.274	9.60E-09	0.937	-0.072	0.049	0.142
rs6575005	14:26954078	T	C	0.758	0.934	0.159	4.40E-09	0.760	0.013	0.028	0.636
rs7115226	11:113408518	A	C	0.074	1.594	0.261	1.70E-09	0.071	-0.001	0.048	0.992

rs72804080	2:59358659	G	A	0.15	1.068	0.192	2.90E-08	0.848	-0.034	0.033	0.302
rs73219758	8:14279446	G	A	0.708	0.984	0.15	5.60E-11	0.287	-0.005	0.025	0.855
rs7503199	17:8134275	C	T	0.734	0.885	0.154	1.00E-08	0.263	0.009	0.026	0.726
rs75539574	2:58871658	C	A	0.086	2.175	0.244	6.90E-19	0.911	-0.014	0.042	0.740
rs7556815	2:114085785	A	G	0.219	2.443	0.164	1.30E-49	0.225	-0.007	0.029	0.814
rs7616632	3:137031237	T	G	0.522	0.792	0.136	4.30E-09	0.525	0.015	0.023	0.506
rs7644809	3:107564459	T	C	0.422	0.784	0.138	1.60E-08	0.428	-0.043	0.024	0.070
rs7806045	7:132610266	T	C	0.755	0.887	0.158	1.40E-08	0.753	-0.032	0.027	0.228
rs7915425	10:125016501	T	C	0.175	1.144	0.179	2.00E-10	0.186	0.022	0.030	0.477
rs7951019	11:118358027	G	T	0.032	2.213	0.391	1.20E-08	0.969	-0.132	0.079	0.093
rs80193650	6:33464363	G	A	0.162	1.01	0.184	4.10E-08	0.839	-0.031	0.033	0.350
rs8038326	15:47989799	A	G	0.727	0.955	0.152	2.80E-10	0.719	-0.055	0.025	0.031
rs8050478	16:56120461	G	A	0.5	0.96	0.136	1.70E-12	0.495	0.040	0.023	0.090
rs915416	1:34731984	C	G	0.29	1.156	0.15	9.90E-15	0.293	0.023	0.026	0.360
rs9345234	6:93162639	C	A	0.578	0.781	0.138	1.80E-08	0.432	0.033	0.023	0.155
rs9382445	6:54937974	T	C	0.623	0.872	0.14	4.80E-10	0.634	-0.013	0.025	0.593
rs9903973	17:50571227	C	T	0.467	0.766	0.136	2.60E-08	0.537	0.020	0.023	0.380
rs9940646	16:53800629	C	G	0.578	1.017	0.137	1.20E-13	0.569	-0.020	0.023	0.398

Note1: EA, effect allele; OA, other allele; EAF, effect allele frequency.

Note2: ICH data not included.

Table S10. SNP Proxies Utilized in the MR analyses of Sleep and ICH

Sleep Traits	Initial SNP associated with Sleep Traits						Proxied SNPs in ICH Datasets						R^2	D'
	RSID	CHR	POS	REF	ALT	MAF	RSID	CHR	POS	REF	ALT	MAF		
Short Sleep	rs1229762	7	114218582	C	T	0.376	rs12705966	7	114248851	A	G	0.372	0.983	-1.000
	rs12567114	1	98527951	G	A	0.278	rs7549825	1	98554409	A	G	0.298	0.862	0.975
	rs3776864	5	102327868	A	C	0.300	rs11955683	5	102326696	A	G	0.300	1.000	1.000
Long Sleep	rs17688916	17	43778680	A	T	0.206	rs113871181	17	43852733	G	A	0.225	0.898	0.988
Sleep Duration	rs34731055	7	2106928	C	T	0.210	rs4470910	7	2071723	C	T	0.213	0.982	1.000
	rs10973207	9	37100525	G	T	0.147	rs62535668	9	37098306	C	G	0.147	1.000	1.000
	rs1517572	11	28829882	A	C	0.407	rs1517564	11	28840418	T	G	0.425	0.925	1.000
	rs10483350	14	29816155	A	G	0.250	rs77493530	14	29855153	C	T	0.215	0.818	1.000
	rs12567114	1	98527951	G	A	0.278	rs7549825	1	98554409	A	G	0.298	0.862	0.975
	rs10421649	19	9942262	T	A	0.455	rs3786694	19	9955341	T	C	0.457	0.953	0.980
	rs151014368	5	176751059	G	A	0.226	rs67008484	5	176735383	T	C	0.249	0.820	0.965
	rs180769	5	135615615	T	C	0.412	rs180767	5	135618719	G	A	0.385	0.878	0.991

Note1: European population panel, 1000 Genomes Phase3.
2: CHR, chromosome.
3: POS, position, GRCH37 build.
4: MAF, minor allele frequency.

Table S11. Harmonised Datasets for the MR Analyses of Short Sleep on ICH

SNP	Chr:Pos	EA	OA	Association with Short Sleep				ICH				Lobar ICH				Non-Lobar ICH			
				EAF	Beta	Se	Pval	EAF	Beta	Se	Pval	EAF	Beta	Se	Pval	EAF	Beta	Se	Pval
rs2863957	2:114089551	A	C	0.218	-0.054	0.007	2.60E-18	0.226	-0.065	0.061	0.290	0.229	-0.066	0.080	0.405	0.230	-0.064	0.073	0.379
rs1229762	7:114218582	T	C	0.665	0.037	0.006	1.10E-12	0.640	-0.054	0.053	0.316	0.640	-0.096	0.070	0.173	0.646	-0.011	0.064	0.858
rs1380703	2:57941287	A	G	0.616	-0.035	0.006	1.60E-11	0.631	-0.024	0.053	0.644	0.632	-0.036	0.069	0.602	0.632	-0.002	0.063	0.973
rs4585442	5:135508381	A	G	0.689	-0.031	0.006	8.10E-10	0.708	-0.049	0.057	0.396	0.708	-0.052	0.074	0.483	0.713	-0.037	0.069	0.591
rs2820313	1:201870221	A	G	0.659	-0.031	0.006	2.30E-09	0.688	0.052	0.055	0.348	0.687	0.032	0.073	0.665	0.684	0.019	0.065	0.772
rs17005118	4:82288564	A	G	0.265	0.030	0.007	2.50E-09	0.263	0.022	0.057	0.701	0.260	-0.029	0.075	0.703	0.264	0.041	0.069	0.550
rs5757675	22:39838892	T	G	0.740	-0.034	0.007	2.70E-09	0.706	0.117	0.057	0.038	0.701	0.065	0.074	0.382	0.707	0.158	0.068	0.021
rs12567114	1:98527951	A	G	0.725	0.036	0.007	4.10E-09	0.691	-0.077	0.057	0.176	0.693	-0.096	0.074	0.194	0.697	-0.034	0.067	0.610
rs2186122	1:66470206	A	T	0.438	-0.024	0.006	4.80E-09	0.435	-0.143	0.052	0.006	0.452	-0.062	0.067	0.353	0.433	-0.201	0.062	0.001
rs11763750	7:2080114	A	G	0.186	-0.035	0.008	5.10E-09	0.212	-0.005	0.062	0.941	0.211	-0.003	0.081	0.974	0.209	-0.030	0.075	0.686
rs9367621	6:55040290	A	T	0.569	-0.024	0.006	1.60E-08	0.578	-0.066	0.052	0.205	0.578	-0.092	0.067	0.169	0.582	-0.064	0.062	0.302
rs3776864	5:102327868	A	C	0.667	0.031	0.006	1.70E-08	0.682	0.094	0.055	0.087	0.679	0.097	0.071	0.169	0.681	0.098	0.065	0.133
rs60882754	8:52886619	A	T	0.939	0.055	0.012	1.80E-08	0.943	-0.161	0.112	0.151	0.945	-0.207	0.146	0.155	0.943	-0.177	0.133	0.184
rs59779556	16:56227965	T	G	0.554	0.025	0.006	2.00E-08	0.548	0.048	0.051	0.350	0.550	0.077	0.066	0.242	0.541	0.011	0.061	0.856
rs205024	17:11227352	T	C	0.383	-0.031	0.006	2.70E-08	0.407	0.064	0.052	0.219	0.401	0.030	0.069	0.668	0.409	0.096	0.062	0.122
rs12661667	6:41792545	T	C	0.263	0.028	0.007	2.80E-08	0.248	0.032	0.060	0.589	0.250	0.096	0.076	0.209	0.244	-0.039	0.072	0.589
rs9321171	6:129848635	T	C	0.460	-0.031	0.006	4.20E-08	0.462	-0.026	0.052	0.610	0.466	0.006	0.067	0.930	0.462	-0.037	0.062	0.543
rs7524118	1:34736052	T	C	0.292	-0.030	0.006	4.90E-08	0.308	-0.010	0.056	0.859	0.311	0.001	0.072	0.990	0.308	-0.004	0.066	0.956

Note1: EA, effect allele; OA, other allele; EAF, effect allele frequency.

Table S12. Harmonised Datasets for the MR Analyses of Long Sleep on ICH

SNP	Chr:Pos	EA	OA	Association with Long Sleep				ICH				Lobar ICH				Non-Lobar ICH			
				EAF	Beta	Se	Pval	EAF	Beta	Se	Pval	EAF	Beta	Se	Pval	EAF	Beta	Se	Pval
rs6737318	2:114083120	A	G	0.778	-0.076	0.011	3.40E-13	0.774	0.076	0.062	0.219	0.771	0.077	0.080	0.337	0.770	0.077	0.073	0.295
rs17817288	16:53807764	A	G	0.518	0.039	0.009	8.90E-09	0.511	0.005	0.051	0.924	0.514	0.013	0.067	0.847	0.510	-0.004	0.061	0.946
rs3751046	11:122828342	A	G	0.853	-0.070	0.013	2.00E-08	0.853	-0.062	0.073	0.401	0.855	-0.004	0.097	0.968	0.852	-0.088	0.086	0.307
rs10899257	11:76415209	A	G	0.144	0.068	0.013	4.60E-08	0.147	-0.100	0.073	0.169	0.150	-0.101	0.093	0.278	0.149	-0.094	0.086	0.276
rs113871181	17: 43852733	A	G	0.204	-0.071	0.012	1.10E-11	0.233	0.063	0.061	0.301	0.236	0.113	0.079	0.154	0.230	0.040	0.073	0.583

Note1: EA, effect allele; OA, other allele; EAF, effect allele frequency.

Table S13. Harmonised Datasets for the MR Analyses of Sleep Duration on ICH

SNP	Chr:Pos	EA	OA	Association with Sleep Duration				ICH				Lobar ICH				Non-Lobar ICH			
				EAF	Beta	Se	Pval	EAF	Beta	Se	Pval	EAF	Beta	Se	Pval	EAF	Beta	Se	Pval
rs7556815	2:114085785	a	g	0.219	2.44	0.16	1.30E-49	0.226	-0.068	0.062	0.267	0.229	-0.074	0.080	0.357	0.230	-0.065	0.073	0.370
rs12607679	18:53059748	t	c	0.738	1.21	0.16	8.30E-15	0.735	-0.096	0.058	0.097	0.742	-0.068	0.077	0.372	0.734	-0.135	0.068	0.046
rs915416	1:34731984	c	g	0.290	1.16	0.15	9.90E-15	0.307	-0.006	0.056	0.922	0.309	0.000	0.073	0.998	0.307	0.013	0.066	0.844
rs9940646	16:53800629	c	g	0.578	1.02	0.14	1.20E-13	0.571	0.004	0.052	0.935	0.574	-0.005	0.069	0.937	0.570	-0.002	0.061	0.969
rs13109404	4:102896591	t	g	0.928	1.87	0.26	1.40E-12	0.929	0.079	0.105	0.453	0.928	0.133	0.138	0.337	0.929	0.109	0.123	0.375
rs8050478	16:56120461	a	g	0.500	-0.96	0.14	1.70E-12	0.498	0.010	0.051	0.852	0.501	0.048	0.066	0.470	0.492	-0.024	0.061	0.689
rs56372231	5:102321905	t	c	0.334	1.02	0.14	2.20E-12	0.317	-0.092	0.055	0.094	0.321	-0.096	0.071	0.175	0.319	-0.096	0.065	0.141
rs13088093	3:135838598	t	g	0.664	-0.98	0.14	7.00E-12	0.668	-0.034	0.054	0.530	0.669	0.028	0.071	0.694	0.668	-0.050	0.063	0.431
rs3095508	16:6550400	a	c	0.406	-0.92	0.14	3.10E-11	0.381	0.045	0.052	0.391	0.378	-0.025	0.069	0.720	0.384	0.068	0.062	0.269
rs34731055	7:2106928	t	c	0.181	1.17	0.18	3.70E-11	0.206	0.015	0.063	0.814	0.205	0.011	0.083	0.894	0.203	-0.016	0.076	0.834
rs73219758	8:14279446	a	g	0.292	-0.98	0.15	5.60E-11	0.293	-0.026	0.056	0.641	0.294	-0.031	0.072	0.662	0.296	-0.015	0.066	0.826
rs10973207	9:37100525	g	t	0.842	-1.23	0.19	6.00E-11	0.830	0.021	0.069	0.766	0.830	-0.015	0.089	0.866	0.831	0.058	0.082	0.482
rs4592416	11:43800474	a	g	0.536	-0.88	0.14	9.30E-11	0.555	0.043	0.052	0.414	0.553	-0.006	0.067	0.930	0.557	0.068	0.062	0.270
rs365663	5:1428883	a	g	0.546	0.88	0.14	1.00E-10	0.538	0.010	0.051	0.848	0.538	0.018	0.068	0.787	0.537	0.020	0.061	0.743
rs1517572	11:28829882	a	c	0.419	-0.88	0.14	1.50E-10	0.446	-0.003	0.051	0.950	0.447	0.014	0.067	0.831	0.443	-0.022	0.061	0.716
rs7915425	10:125016501	t	c	0.175	1.14	0.18	2.00E-10	0.199	-0.013	0.065	0.848	0.198	-0.025	0.086	0.771	0.198	-0.048	0.077	0.534
rs330088	8:9149746	t	c	0.453	-0.87	0.14	2.70E-10	0.470	0.014	0.053	0.785	0.464	-0.036	0.068	0.596	0.474	0.046	0.062	0.458
rs8038326	15:47989799	a	g	0.727	0.96	0.15	2.80E-10	0.720	-0.036	0.057	0.522	0.719	-0.047	0.074	0.526	0.719	-0.037	0.067	0.581

rs9382445	6:54937974	t	c	0.623	0.87	0.14	4.80E-10	0.632	-0.032	0.053	0.550	0.632	-0.092	0.070	0.186	0.638	-0.004	0.063	0.949
rs4767550	12:117951150	a	g	0.586	-0.86	0.14	6.30E-10	0.593	0.000	0.052	0.996	0.587	-0.061	0.067	0.362	0.600	0.064	0.062	0.302
rs1057703	11:122830251	t	g	0.853	-1.16	0.19	1.10E-09	0.853	-0.061	0.073	0.406	0.855	-0.002	0.097	0.984	0.852	-0.085	0.086	0.322
rs4128364	2:147612734	t	c	0.661	-0.88	0.14	1.40E-09	0.660	-0.088	0.055	0.105	0.669	-0.012	0.071	0.866	0.661	-0.130	0.065	0.045
rs61796569	1:66476437	t	c	0.270	0.93	0.15	1.50E-09	0.257	-0.160	0.059	0.007	0.268	-0.100	0.076	0.188	0.259	-0.181	0.071	0.010
rs10483350	14:29816155	g	a	0.195	1.04	0.17	1.50E-09	0.184	0.035	0.067	0.601	0.182	-0.002	0.089	0.980	0.184	0.080	0.080	0.316
rs7115226	11:113408518	a	c	0.074	1.59	0.26	1.70E-09	0.075	0.068	0.099	0.492	0.074	0.070	0.130	0.591	0.074	0.087	0.120	0.470
rs269054	1:57864304	a	t	0.422	0.82	0.14	2.10E-09	0.427	-0.024	0.052	0.649	0.423	-0.052	0.069	0.453	0.431	0.028	0.062	0.653
rs112230981	3:55879269	a	g	0.950	1.89	0.31	2.20E-09	0.961	0.162	0.140	0.248	0.958	0.073	0.179	0.683	0.960	0.216	0.164	0.189
rs11602180	11:48162453	t	c	0.163	-1.10	0.18	2.30E-09	0.183	-0.102	0.067	0.130	0.186	-0.094	0.087	0.278	0.185	-0.111	0.080	0.165
rs2192528	4:18327896	a	g	0.480	0.80	0.14	2.70E-09	0.454	0.049	0.052	0.342	0.456	0.116	0.067	0.085	0.446	0.008	0.061	0.895
rs205024	17:11227352	t	c	0.384	0.83	0.14	3.90E-09	0.407	0.064	0.052	0.219	0.401	0.030	0.069	0.668	0.409	0.096	0.062	0.122
rs12567114	1:98527951	g	a	0.724	-0.89	0.15	4.30E-09	0.691	-0.077	0.057	0.176	0.693	-0.096	0.074	0.194	0.697	-0.034	0.067	0.610
rs7616632	3:137031237	t	g	0.522	0.79	0.14	4.30E-09	0.531	0.081	0.051	0.111	0.532	0.114	0.067	0.090	0.524	0.022	0.060	0.714
rs6575005	14:26954078	t	c	0.758	0.93	0.16	4.40E-09	0.756	-0.020	0.060	0.738	0.760	0.069	0.081	0.399	0.754	-0.049	0.070	0.477
rs11621908	14:78495761	t	c	0.083	-1.45	0.25	5.60E-09	0.092	0.074	0.089	0.404	0.089	-0.039	0.120	0.743	0.093	0.109	0.105	0.300
rs10421649	19:9942262	t	a	0.443	-0.80	0.14	6.90E-09	0.478	-0.044	0.052	0.399	NA	NA	NA	NA	0.481	-0.026	0.061	0.671
rs2072727	20:43538733	t	c	0.436	0.80	0.14	7.90E-09	0.402	-0.133	0.052	0.011	0.416	-0.051	0.068	0.455	0.401	-0.195	0.062	0.002
rs113113059	6:43160375	t	c	0.780	0.97	0.16	8.40E-09	0.780	-0.067	0.062	0.280	0.788	0.072	0.083	0.384	0.777	-0.106	0.073	0.146
rs374153	2:40382712	t	c	0.842	-1.06	0.19	9.10E-09	0.848	-0.031	0.071	0.662	0.852	0.026	0.094	0.785	0.846	-0.079	0.085	0.348
rs151014368	5:176751059	g	a	0.794	-0.97	0.17	9.10E-09	0.757	0.047	0.061	0.435	0.754	0.051	0.078	0.514	0.757	0.072	0.072	0.318
rs62120041	2:9185564	t	c	0.934	1.57	0.27	9.60E-09	0.930	-0.172	0.102	0.091	0.933	-0.176	0.132	0.184	0.932	-0.139	0.120	0.249
rs7503199	17:8134275	t	c	0.266	-0.89	0.15	1.00E-08	0.265	-0.083	0.058	0.149	0.271	0.003	0.075	0.968	0.265	-0.103	0.069	0.134

rs17732997	3:70470834	c	g	0.569	0.78	0.14	1.20E-08	0.581	-0.001	0.051	0.982	0.580	0.014	0.067	0.832	0.579	-0.021	0.061	0.733
rs61985058	14:60233841	t	c	0.143	1.12	0.19	1.30E-08	0.127	0.144	0.078	0.063	0.129	0.256	0.097	0.009	0.120	0.004	0.094	0.967
rs17427571	4:82254908	a	g	0.684	0.83	0.15	1.30E-08	0.676	0.029	0.055	0.600	0.676	0.027	0.071	0.707	0.676	0.035	0.065	0.591
rs7806045	7:132610266	t	c	0.755	0.89	0.16	1.40E-08	0.767	0.000	0.061	0.998	0.762	-0.049	0.079	0.536	0.768	0.033	0.073	0.649
rs35531607	4:92533225	t	c	0.526	-0.77	0.14	1.50E-08	0.533	0.017	0.051	0.744	0.533	0.032	0.066	0.622	0.531	-0.010	0.060	0.866
rs55658675	14:65554638	t	c	0.355	-0.79	0.14	2.00E-08	0.344	0.033	0.054	0.539	0.342	0.048	0.071	0.499	0.342	0.044	0.064	0.490
rs11567976	5:137654218	t	c	0.571	0.77	0.14	2.10E-08	0.533	0.011	0.052	0.838	0.530	-0.012	0.067	0.859	NA	NA	NA	NA
rs180769	5:135615615	c	t	0.575	-0.76	0.14	2.30E-08	0.597	-0.003	0.053	0.949	0.601	0.041	0.070	0.556	0.595	-0.024	0.063	0.697
rs1553132	11:88297740	a	g	0.742	-0.87	0.16	2.50E-08	0.752	0.108	0.059	0.065	0.744	0.024	0.076	0.754	0.752	0.168	0.071	0.018
rs9903973	17:50571227	t	c	0.533	-0.77	0.14	2.60E-08	0.529	0.068	0.051	0.180	0.526	0.042	0.066	0.527	0.529	0.092	0.060	0.127
rs11614986	12:110007939	a	g	0.821	0.98	0.18	2.70E-08	0.827	0.023	0.069	0.741	0.827	0.055	0.090	0.542	0.826	-0.001	0.081	0.990
rs2231265	6:89790201	a	g	0.228	-0.90	0.16	2.70E-08	0.232	0.003	0.061	0.964	0.237	0.076	0.080	0.339	0.228	-0.029	0.074	0.690
rs174560	11:61581764	t	c	0.686	-0.82	0.15	2.80E-08	0.707	-0.095	0.057	0.095	0.711	-0.055	0.075	0.466	0.707	-0.128	0.068	0.058
rs10173260	2:210377845	t	c	0.394	-0.77	0.14	2.90E-08	0.385	-0.069	0.053	0.195	0.385	-0.083	0.069	0.232	0.386	-0.065	0.063	0.301
rs12611523	2:139195328	a	g	0.545	0.76	0.14	3.10E-08	0.549	0.041	0.052	0.431	0.548	0.056	0.069	0.416	0.547	0.047	0.062	0.443
rs11643715	16:23909538	c	g	0.709	-0.83	0.15	3.20E-08	0.687	-0.032	0.056	0.567	0.685	-0.101	0.074	0.170	0.691	0.010	0.067	0.880
rs4538155	2:157040773	t	c	0.647	0.78	0.14	3.60E-08	0.643	0.020	0.054	0.711	0.638	-0.020	0.071	0.780	0.646	0.030	0.064	0.635
rs80193650	6:33464363	a	g	0.838	-1.01	0.18	4.10E-08	0.820	0.003	0.068	0.965	0.818	-0.018	0.088	0.841	0.821	0.022	0.081	0.789
rs10761674	10:64618340	t	c	0.523	-0.74	0.14	4.20E-08	0.524	-0.066	0.051	0.194	0.526	-0.076	0.067	0.254	0.527	-0.056	0.061	0.359
rs11190970	10:103128332	a	g	0.201	-0.92	0.17	4.60E-08	0.208	-0.083	0.064	0.192	0.207	-0.151	0.085	0.075	0.213	-0.027	0.074	0.715

Note1: EA, effect allele; OA, other allele; EAF, effect allele frequency.

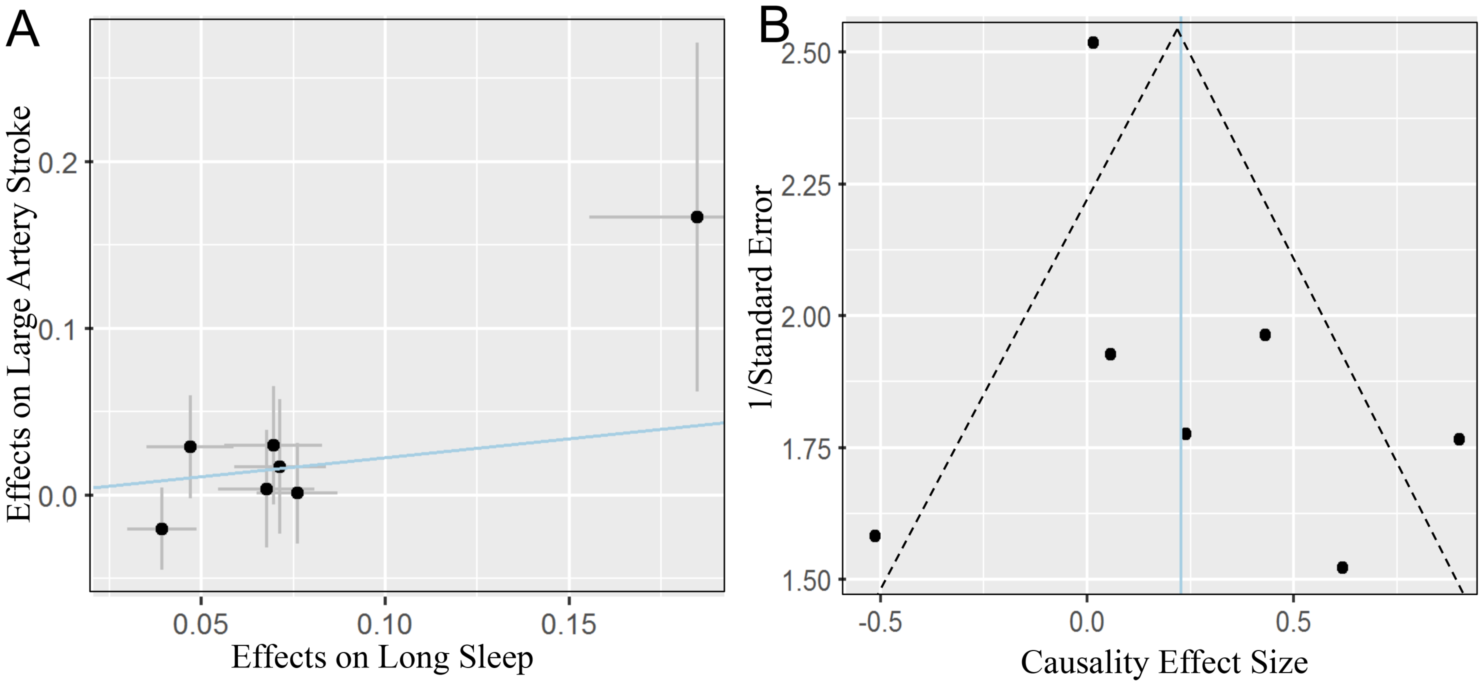

Fig S1. Causal effect of long sleep on large artery stroke by individual SNP and primary overall estimate by Mendelian randomization.
(A) Overall estimate by the inverse-variance weighted method (the fitted line slope), individual SNP effect on large artery stroke (each point and vertical line) against its effect on long sleep (each point and horizontal line). (B) Funnel plot for the causality estimate of individual instrument variable. Each point denotes the inverse standard error against its effect size estimated by Wald ratio method.

.

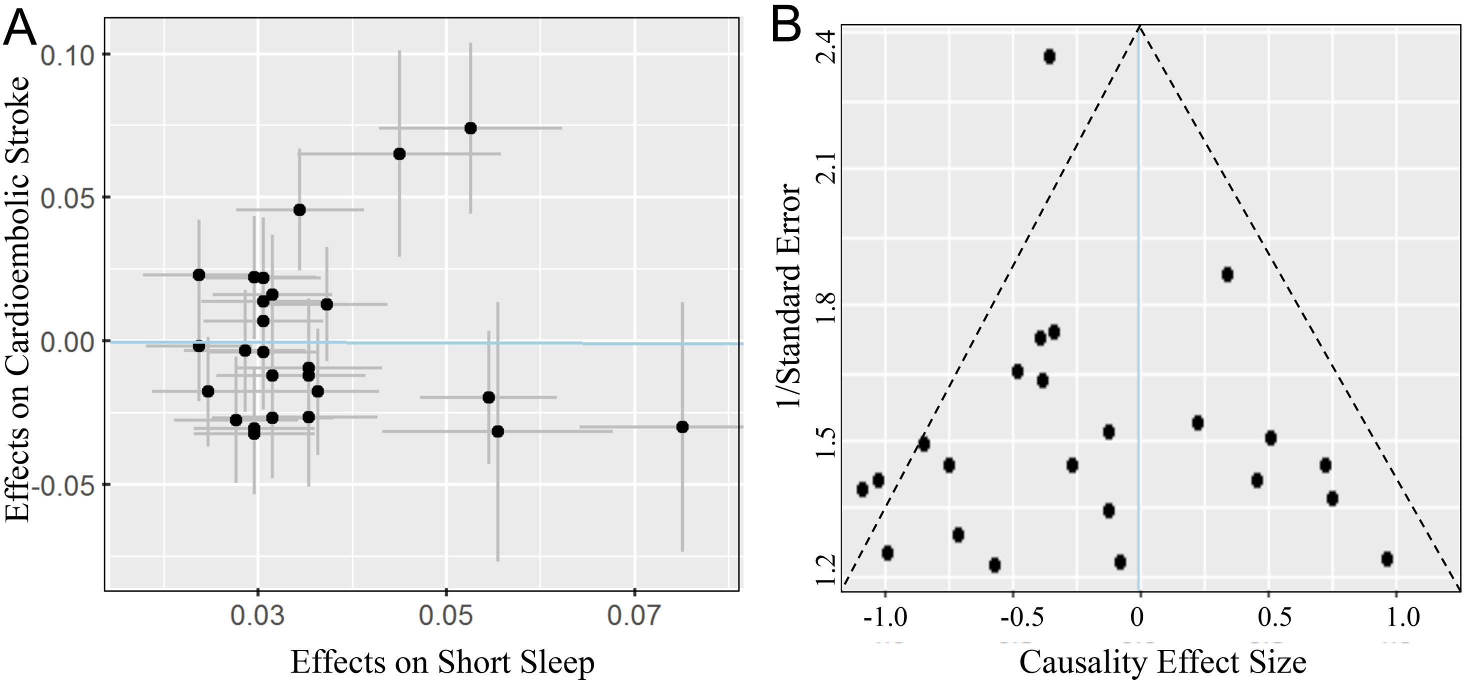

Fig S2. Causal effect of short sleep on cardioembolic stroke by individual SNP primary overall estimate by Mendelian randomization.

(A) Overall estimate by the inverse-variance weighted method (the fitted line slope), individual SNP effect on cardioembolic stroke (each scatter point and vertical line) against its effect on short sleep (each scatter point and horizontal line). (B) Funnel plot for the causality estimate of individual instrument variable. Each point denotes the inverse standard error against its effect size estimated by Wald ratio method.

.

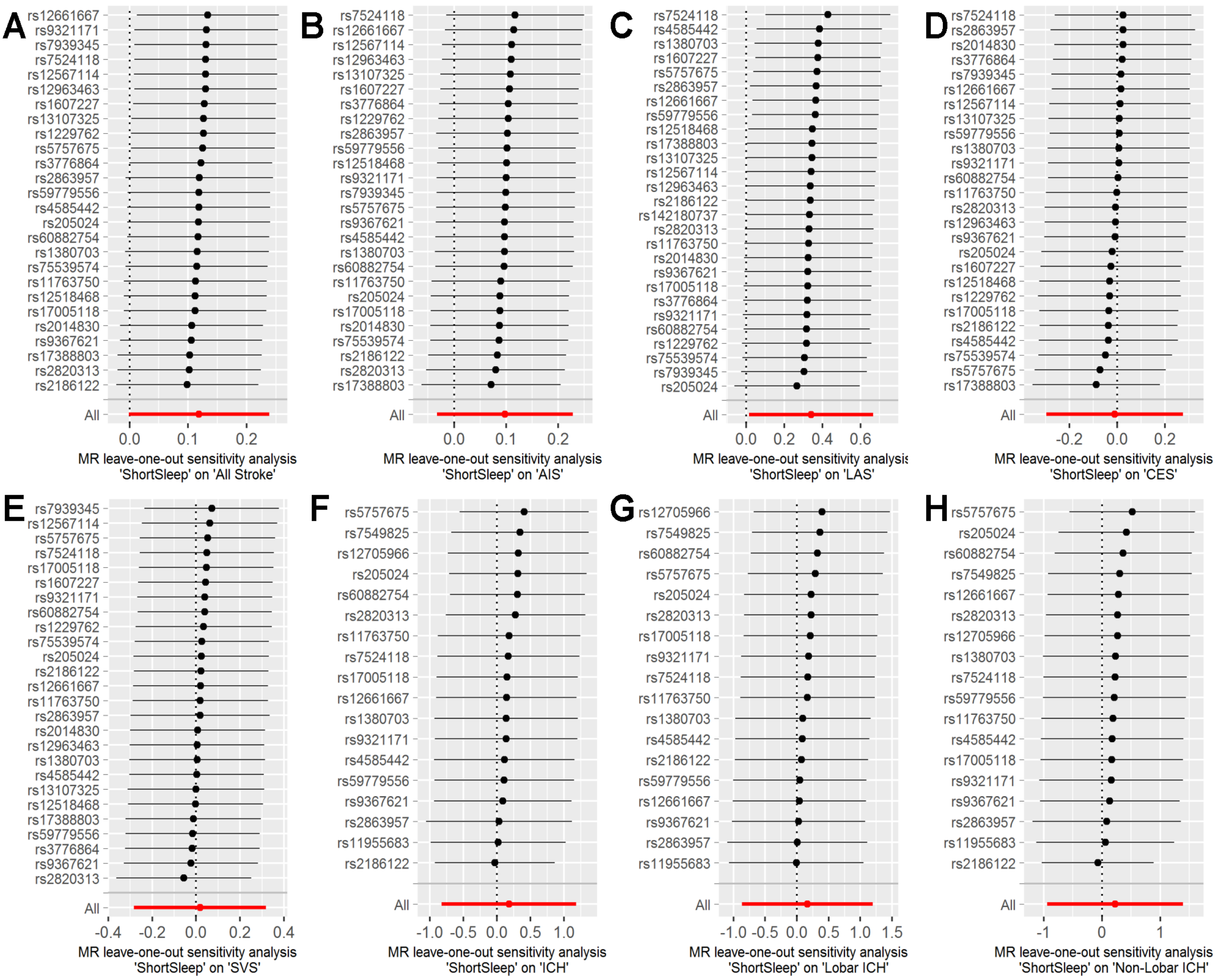

Fig S3. Leave-one-out Plots for the MR Analyses of Short Sleep on Stoke

AIS, any ischemic stroke; CES, cardioembolic stroke; ICH, Intracerebral Hemorrhage; LAS, large artery stroke; SVS, small vessel stroke.

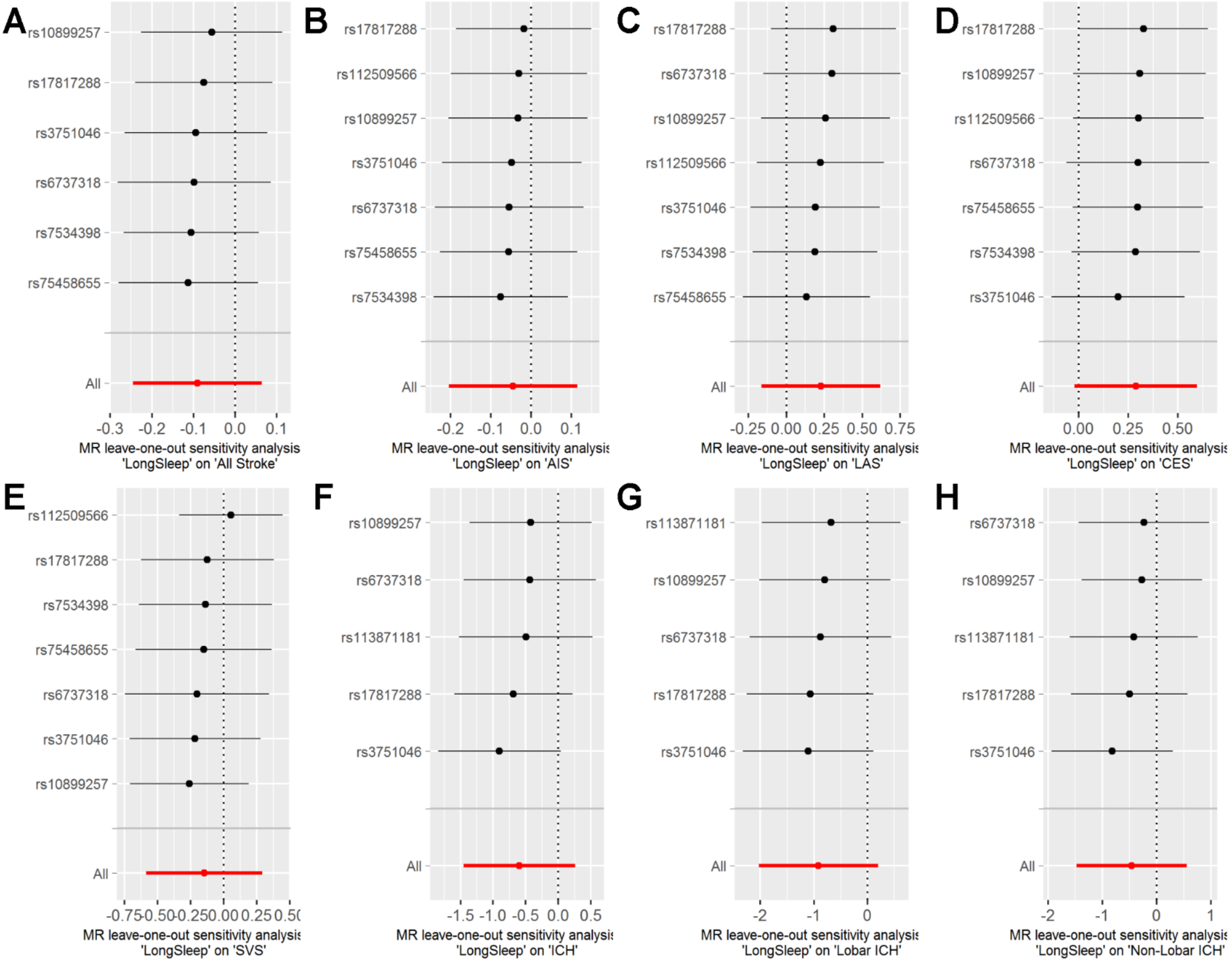

Fig S4. Leave-one-out Plots for the MR Analyses of Long Sleep on Stoke

AIS, any ischemic stroke; CES, cardioembolic stroke; ICH, Intracerebral Hemorrhage; LAS, large artery stroke; SVS, small vessel stroke.

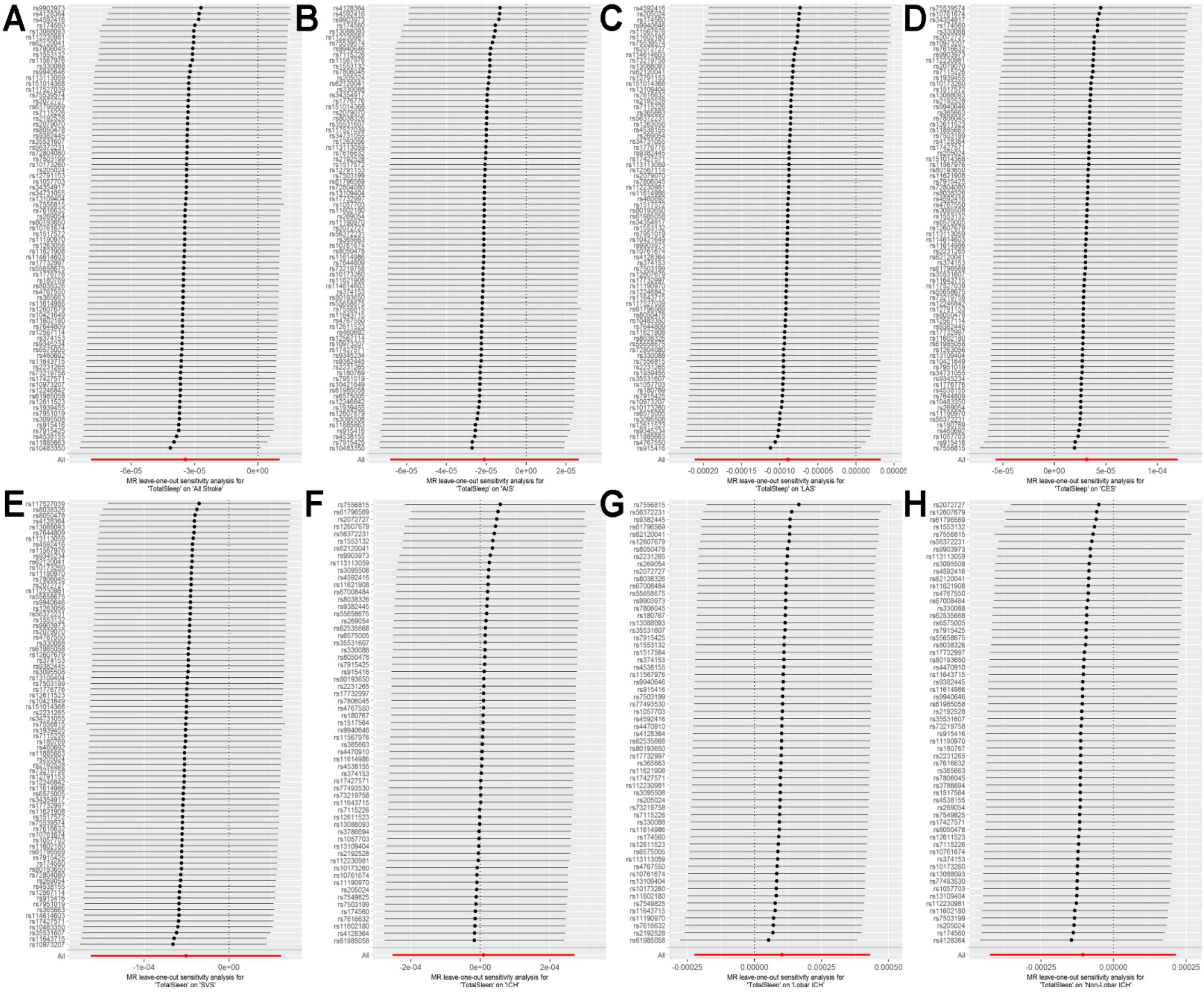

Fig S5. Leave-one-out Plots for the MR Analyses of Sleep Duration on Stoke

AIS, any ischemic stroke; CES, cardioembolic stroke; ICH, Intracerebral Hemorrhage. LAS, large artery stroke; SVS, small vessel stroke.
